# *Rhodosporidium toruloides*—a new surrogate model to study rapamycin induced effects on human aging and cancer

**DOI:** 10.1007/s00018-025-05662-4

**Published:** 2025-04-09

**Authors:** Philipp M. Cavelius, Martina Haack, Dania Awad, Thomas B. Brueck, Norbert Mehlmer

**Affiliations:** Department of Chemistry, Werner Siemens-Chair of Synthetic Biotechnology, Technical University of Munich (TUM), Garching, Germany

**Keywords:** Haplotypes, Rhodosporidium, Rapamycin, Target of rapamycin, Time-resolved proteomics

## Abstract

The haploid, olegenious yeast *Rhodosporidium toruloides* accumulates intracellular lipids and carotenoids upon metabolic stress. Target of Rapamycin (TOR) signaling, essential for cell proliferation, is known to affect cellular lipid accumulation. In contrast to the conventional surrugate cell model *S. cerevisiae*, which harbours two TOR kinases within its TOR complex, *R. toruloides* only harbours one TOR kinase, mimicking mammalian systems. We used a proteomics centered approach to probe the cellular response, of the two *R. toruloides* haplotypes, IFO0559 and IFO0880 upon treatment with the TOR inhibitor rapamycin, with an original focus on difference in carotenoid and lipid accumulation. Unexpectedly, IFO0880 displayed severe growth arrest in response to rapamycin, while IFO0559 did not. Proteomic anaysis revealed differential expression of several proteins involved in cell cycle control, lipogensis, amino acid metabolism and autophagy between the two haplotypes. Among those we identified several proteins previously described in both mammalian oncogenic and aging contexts. This differential haplotype response to rapamycin treatment positions *R. toruloides* as a promising cell surrugate model to study cellular mechanisms underlying rapamycin response especially for systems with high lipid contents, an emerging hallmark of different forms of mammalian cancer and age related disease.

**Supplementary Information:**

The online version contains supplementary material available at 10.1007/s00018-025-05662-4.

## Introduction

The basidiomycete *Rhodosporidium toruloides*, a haploid yeast, is a focus of scientific interest for its capability of accumulating high amounts of carotenoids and lipids, while also capable of metabolizing a broad spectrum of carbon sources, enabling the use of low-cost biomass waste streams [1–3]. In addition, *R. toruloides* is easy to cultivate and exhibits high resistance to different stresses including oxidative stress and toxic compounds [1, 3, 4]. Notably, there are two haplotypes of *R. toruloides*. Members of opposing haplotypes can exhibit vastly different growth and metabolism, which also affects carotenoid and lipid accumulation [3, 5]. However, direct comparisons between those haplotypes is scarce.

This contrasts the well-characterized model yeasts *Saccharomyces cerevisiae*, which has been studied as surrogate model for mammalian systems in different research areas [6, 7]. One of *S. cerevisiae* model functions is in Target of Rapamycin (TOR) signaling. TOR, a serine/threonine kinase of the phosphatidylinositol family, together with a set of other proteins, forms two complexes, Target of Rapamycin Complex 1 and 2 (TORC1 & TORC2) [8, 9]. TORC1 responds to extracellular and intracellular cues, especially the nutritional status. Processes affected by TORC1 signaling include stress response, cell metabolism and autophagy, growth and proliferation as well as survival and apoptosis. Furthermore, TOR signaling impacts translational activity by affecting ribosome biogenesis and different translational factors, such as eukaryotic translation initiation factor 4E (eIF-4E) [8, 10, 11].

The evolutionary conservation of this pathway emphasizes its importance. TOR signaling can be found among most eukaryotes. Therefore, many experiments were performed on *S. cerevisiae* as an easy to handle and fast to perform model organism. In fact, the TOR-pathway was discovered in *S. cerevisiae*. However, the human/mammalian TOR pathway (mTOR) only includes one TOR kinase found in both TORC1 and TORC2. *S. cerevisiae*, on the other hand, expresses two highly similar but distinct kinases for TORC1 and TORC2 [8, 9]. Resembling the mTOR pathway, *R. toruloides* TOR-signaling involves only one TOR kinase to produce TORC1 and TORC2 [12].

An inhibitor of TOR signaling, rapamycin, named after the island of its discovery rapa nui, was initially described in the context of antifungal activity. Current research expands its functions to immunosuppression, anti-aging, and cancer treatment, as TORC1 dysregulation is found in several types of cancer. [8, 9, 13–17].

To evaluate the impact of rapamycin on lipid and carotenoid accumulation, both haplotypes of *R. toruloides* were subjected to 5 μM rapamycin, while lipid and carotenoid levels were monitored at two time points. Additionally, a proteomic approach allowed for evaluation of changes in protein abundance in lipid and carotenoid metabolic pathways.

In this study, we present the comparative data, revealing haplotype-specific differences in their response to rapamycin significantly affecting growth as well as lipid and carotenoid accumulation. The cumulative data presents reduced sensitivity towards rapamycin by one of the haplotypes, manifesting behavior and proteomic changes previously observed in human cancer cells linked to increased cell proliferation and avoidance of apoptosis.

## Results

### Influence of rapamycin on growth

**Fig. 1:**
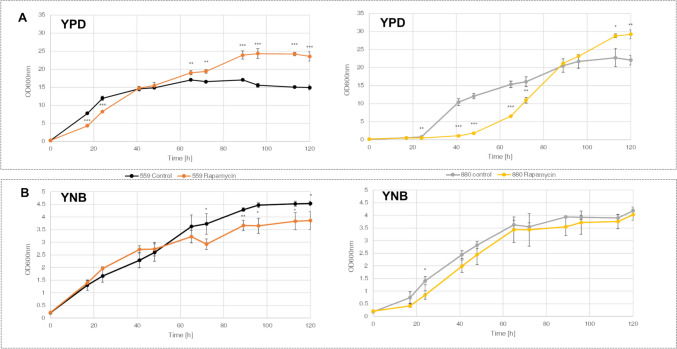
Growth of IFO0559 and IFO0880 with and without addition of rapamycin measured in OD_600nm_ cultivated in **a** YPD and **b** YNB; n = 3, significance of difference between rapamycin treatment and respective control evaluated by t-test *p < 0.05, **p < 0.01, ***p < 0.001

**Fig. 2:**
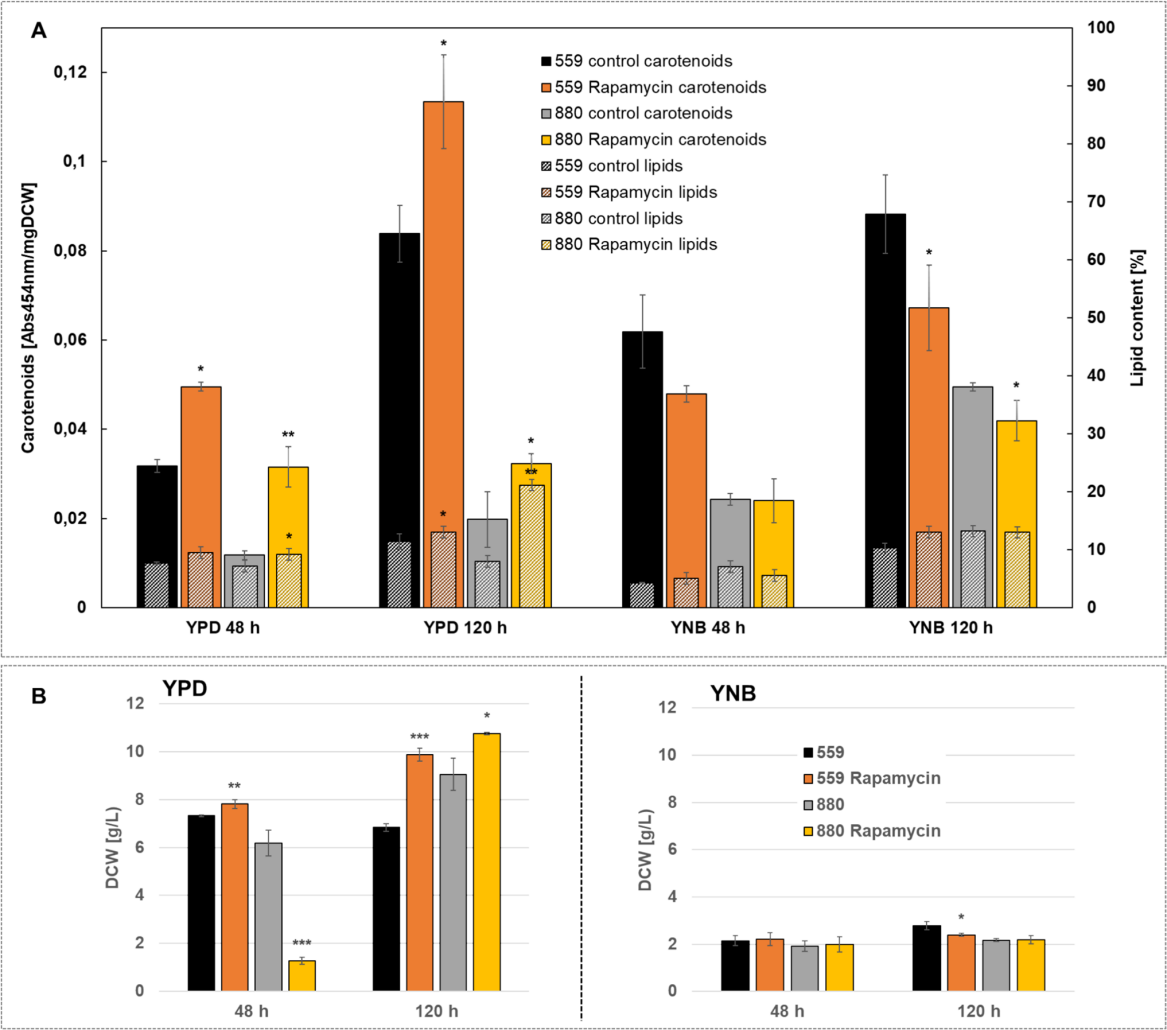
**a** Carotenoid and lipid content normalized to mg DCW for both haplotypes IFO0559 and IFO0880 with and without addition of rapamycin in YPD and YNB at 48 h and 120 h, **b** dry cell weight of IFO0559 and IFO0880 with and without addition of rapamycin in YPD and YNB at 48 h and 120 h; n = 3, significance of difference between rapamycin treatment and respective control evaluated by t-test *p < 0.05, **p < 0.01, ***p < 0.001

Optical density at 600 nm (OD600nm) reveals severe inhibition of growth in IFO0880 cultures, grown in YPD medium in the presence of rapamycin. At 48 h, samples grown without addition of rapamycin exhibit an OD600nm of 12.1, while rapamycin treated samples average at around 1.8. After an additional 24 h, rapamycin treated cultures approximate the OD of untreated cultures and culminate in a final OD600nm of 29.2 at 120 h, surpassing ODs of untreated controls (22.0) (Fig. 1a). Similar results were observed in accumulated dry cell weight (DCW) (Fig. 2b).

In contrast, IFO0559 cultures grown in YPD medium appear almost unaffected by rapamycin treatment, showing only slight delay in growth within the first 24 h (Fig. 1a). Similar to IFO0880, IFO0559 cultures grown in YPD medium and treated with rapamycin exhibit significant increases in final OD600nm and biomass at 120 h (OD600nm of 23.5 comparted to 14.9).

Contrasting the growth effects shown in full medium, YNB grown cultures of both haplotypes exhibit only slight variations from their respective controls upon rapamycin treatment. At 120 h, IFO0559 samples show final ODs of 4.5 and 3.9 and DCW of 2.8 g/l and 2.4 g/l for untreated samples and rapamycin-treated samples, respectively. Similarly, IFO0880 samples exhibit ODs of 4.2 and 4.0 for untreated and rapamycin-treated samples, which is correlated in DCW at 120 h of IFO0559 samples with no significant difference in DCW (around 2.2 g/L).

### Influence of Rapamycin on Catorenoid and Lipid Content

In YNB medium, rapamycin treatment did not increase carotenoid accumulation (Fig. 2). At 48 h, rapamycin treatment led to a decrease from 0.062 Abs454nm/mg to 0.050 Abs454nm/mg in IFO0559 cultures, while IFO0880 samples exhibit no difference in carotenoid content (0.024 Abs454nm/mg). At 120 h, the carotenoid signal drops significantly from 0.088 Abs454nm/mg in untreated IFO0559 samples to an average of 0.067 Abs454nm/mg in rapamycin treated cultures. In IFO0880, a significant decrease from 0.050 Abs454nm/mg to 0.042 Abs454nm/mg is observed. No significant differences in lipid content were observed for both haplotypes upon rapamycin treatment when grown in YNB.

For cultivation in YPD medium, carotenoid levels significantly increased in both haplotypes at 48 h when treated with rapamycin. In IFO0880, carotenoid signal increases by 266.7% from 0.012 Abs454nm/mg to 0.032 Abs454nm/mg. At 120 h carotenoid signal of rapamycin treated samples does not change compared to 48 h, however non-treated samples increase to 0.020 Abs454nm/mg. Similar, IFO0559 cultures exhibit a 156.3% increase in carotenoid content from 0.032 Abs454nm/mg to 0.050 Abs454nm/mg at 48 h upon rapamycin treatment. At 120 h, carotenoid levels of IFO0559 samples not treated with rapamycin reach up to 0.084 Abs454nm/mg with rapamycin-treated samples peaking at 0.113 Abs454nm/mg, measuring a 134.5% increase.

Lipid content is only slightly altered at 48 h from 7.7% to 9.5% and from 7.2% to 9.2% in IFO0559 and IFO0880 respectively, but significantly increases in IFO0880 at 120 h from 8.0% to 21.2%. In IFO0559 cultures, at 120 h, rapamycin led to an increase from 11.4% to 13.0%.

Additionally, fatty acid profiles were analyzed for rapamycin induced changes, however only small shifts were measured across all groups (S2&S6).

### Effect of rapamycin on protein expression

Time-resolved quantitative proteomics enabled deeper insights into cellular regulatory mechanisms active upon treatment with rapamycin. Changes in protein levels between the different groups were evaluated with a cut-off of significance ≥ 2 and fold change ≥ 2 (LFQ by PEAKS Studio). As both haplotypes differ genetically, different databases were used for IFO0559 and IFO0880, making comparisons challenging as proteins might be included in only one of the two databases or might be annotated with a different name.

Upon 48 h of rapamycin treatment in YPD medium, 283 proteins were differentially regulated in IFO0559 cultures, of which 166 proteins were upregulated (Table 1). In IFO0880, 641 proteins were differentially regulated, with 120 being upregulated.

**Table 1:** Summary of protein numbers quantified with significantly different abundance between rapamycin treated samples and control samples comparing the use of both databases corresponding to one of the two haplotypes

Time point	IFO0559 database						IFO0880 database					
	48 h			120 h			48 h			120 h		
Strain and medium	Up	Down	Total	Up	Down	Total	Up	Down	Total	Up	Down	Total
IFO0559 YPD	Count 166	Count 177		Count 423	Count 281		Count 123	Count 110		Count 443	Count 262	
	Avg 3.63	Avg 0.37	**283**	Avg 4.93	Avg 0.35	**704**			**233**			**705**
IFO0559 YNB	Count 156	Count 125		Count 583	Count 13		Count 102	Count 105		Count 489	Count 12	
	Avg 3.68	Avg 0.34	**281**	Avg 4.29	Avg 0.33	**596**			**207**			**501**
IFO0880 YPD	Count 83	Count 513		Count 318	Count 332		Count 120	Count 521		Count 287	Count 464	
			**596**			**650**	Avg 7.85	Avg 0.30	**641**	Avg 3.90	Avg 0.31	**751**
IFO0880 YNB	Count 110	Count 290		Count 640	Count 32		Count 121	Count 354		Count 717	Count 43	
			**400**			**672**	Avg 5.34	Avg 0.33	**475**	Avg 3.35	Avg 0.31	**760**

Only proteins with significance and fold change ≥ 2 are represented. Additionally, for proteins identified with their respective haplotype specific database, average fold-changes are presented

Highlight in bold was only for clearity purpose and must not necessarily be maintained

When cultured in YNB, a similar number of proteins were upregulated at 48 h (IFO0559: 156, IFO0880: 121) with a total of 125 and 354 proteins downregulated in IFO0559 and IFO0880, respectively. In IFO0880, total number of differentially produced proteins decreased in YNB compared to YPD samples at 48 h, while in IFO0559, the numbers of proteins affected was almost identical.

At 120 h, independent of haplotypes and the medium used for cultivation, the amount of differentially regulated proteins increased, with IFO0559, exhibiting 704 and 596 differentially regulated proteins in YPD and YNB, respectively. In IFO0880, 751 and 760 proteins were differentially produced in YPD and YNB, respectively. Notably, YNB cultivated samples exhibited significantly more upregulated proteins than downregulated proteins at 120 h. Furthermore, differences in total number of differentially produced proteins between YPD and YNB grown cultures decreased at 120 h.

In general, the number of proteins affected by rapamycin treatment was higher in IFO0880 than in IFO0559. Additionally, analysis of average fold changes revealed the magnitudes, by which protein levels were impacted in early stages of cultivation (48 h), to be significantly greater in IFO0880 samples (Table 1).

To evaluate whether differences in numbers between IFO0559 and IFO0880 was impacted by the 2 databases used, each sample was additionally analyzed with the opposite database and numbers of differentially produced proteins were determined (Table 1). For most conditions, the number of proteins identified decreased with use of the database cataloging proteins of the opposite haplotype. However, in all cases, numbers were still comparable and trends in differences were preserved.

Functional annotation and analysis revealed significant differential regulation of protein production for proteins involved in the TOR pathway (1), apoptosis, cell cycle regulation and cell proliferation (2) as well as ribosome biogenesis and ribosomal proteins (3) as these processes are known targets of rapamycin treatment.

TOR signaling is strongly affected by amino acid availability, therefore proteins with known impact on free amino acid levels (4) including autophagy, endocytosis, phagocytosis and proteolysis (5) were investigated. These processes might contribute to an adaption to rapamycin treatment by increasing intracellular levels of free amino acids especially arginine as previously demonstrated in *S. cerevisiae* [18].

Finally, differential production of proteins involved in fatty acid and carotenoid metabolism (6) were analyzed.

To simplify evaluation, proteins upregulated by rapamycin treatment are indicated with a fold change greater 1, downregulated proteins with a fold change smaller 1. A fold change of 0.5, therefore is equivalent to a twofold downregulation. Significance and fold change ≥ 2 were chosen as cut-off.

#### TOR Signaling

Several proteins of the TOR signaling pathway were identified in the proteomics data (Table 2, S7&S8). In correlation to the observed growth behavior, no protein of TOR signaling exhibited significant differential regulation in IFO0559 at 48 h when cultured in YPD, while 5 proteins were downregulated in rapamycin treated IFO0880. Those being the protein transporter SEC13 (0.32-fold), 2 different v-type proton ATPase subunits (0.39- and 0.37-fold), likely involved in amino acid sensing and subsequent activation of TORC1 [19], and 2 versions of the translation initiation factor 4E (0.46- and 0.2-fold), an important downstream target for TOR signaling [8, 10]. SEC13, as part of GATOR2, also functions in amino acid sensing and subsequent regulation of TORC1 activity [20]. Furthermore, v-type ATPases are connected to the lysosome, where the import of H + and subsequent reduction of pH activates lysosomal hydrolyzing enzymes for proteolysis. The pH gradient resulting from v-ATPase activity is also involved in the export of amino acids into the cytosol. Due to their various roles, v-ATPases were investigated in context of cancer and drug resistance [21].

**Table 2:**
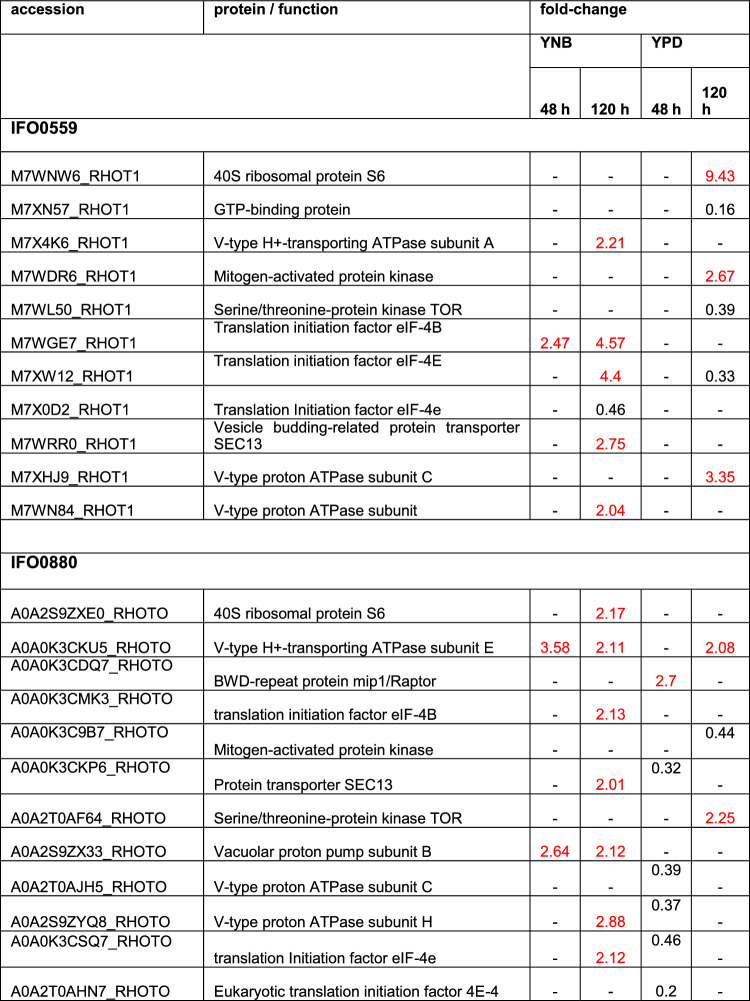
Discussed differentially regulated proteins of the TOR pathway

Proteins depicted with a fold change of 2 and higher are upregulated (marked in red), while proteins with a fold change of 0.5 and lower are downregulated. Coditions in which no significant change was observed are marked by “- “. Fold change and significances of ≥ 2 were used as cut-off

In contrast to YPD groups at 48 h, 3 v-type proton pump subunits and eIEF-4E exhibited increased level when IFO0880 was cultured in YNB for 120 h (2.11-fold, 2.12-fold, 2.88-fold and 2.12-fold, respectively). Similarly, 2 v-type proton ATPase subunit and 2 versions of eIF-4E were identified in YNB grown IFO0559 cultures at 120 h, showing 2.21-, 2.11-, 4.4- and 0.46-fold change in protein abundance.

Protein transporter SEC13 was upregulated upon rapamycin treatment in both haplotypes when grown in YNB for 120 h (2.75-fold and 2.01-fold for IFO0559 and IFO0880, respectively).

The *R. toruloides* TOR kinase was differentially regulated in both haplotypes when cultured in YPD at 120 h. Interestingly, IFO0559 exhibited significant downregulation (0.39-fold), while IFO0880 showed upregulation of 2.25-fold, mirroring the trends observed in growth data.

The 40S ribosomal protein S6, a target of ribosomal protein S6 kinase was significantly upregulated in YPD samples of IFO0559 at 120 h (9.43-fold).

Translation initiation factor eIF-4B, another target of ribosomal protein S6 kinase was upregulated in both IFO0559 and IFO0880 samples when cultured in YNB for 120 h.

#### Apoptosis, cell cycle, cell proliferation

Growth data revealed a significant impact of rapamycin treatment on biomass accumulation, which is in accordance with literature on rapamycin treatment in yeast [18, 22]. Therefore, proteins with impact on growth, apoptosis, cell cycle regulation and cell proliferation were identified and analyzed within the proteomic data.

For IFO0559, 36 distinct proteins involved in these processes were differentially expressed upon rapamycin exposure (Table 3, S7&S8). In YNB samples at 120 h, the S-phase kinase-associated protein 1, an essential part of the SCF E3 ubiquitin ligase complex, and the DNA replication licensing factor MCM3, both involved in cell proliferation and cycle regulation [23–25], were significantly upregulated with 37.77-fold and 28.25-fold upregulation, respectively. However, besides these two proteins, few proteins were affected in YNB grown samples, with most proteins affected at 120 h of cultivation. Only 3 proteins were differentially expressed at 48 h of YNB cultivation, one of which is the translation initiation factor eIF-5, which was also upregulated at 120 h. eIF-5 overexpression was previously hypothesized to help in cell survival [26].

**Table 3:**
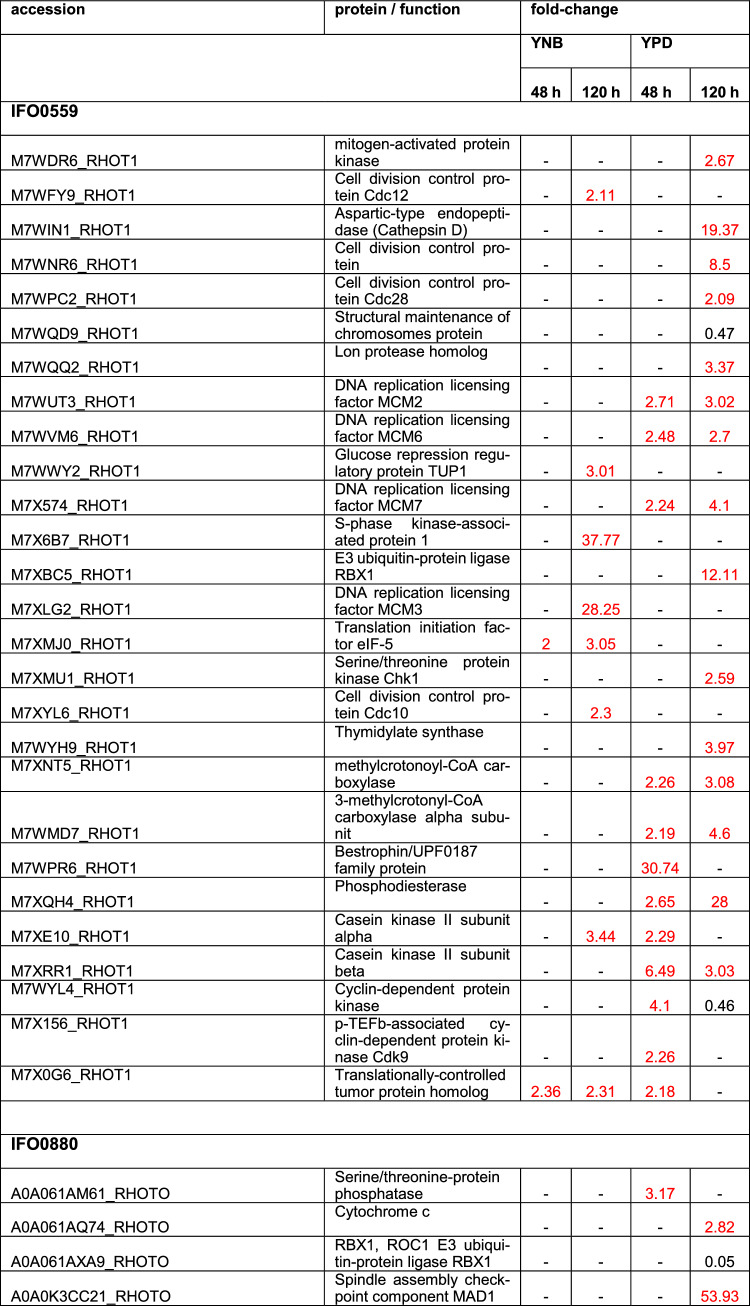

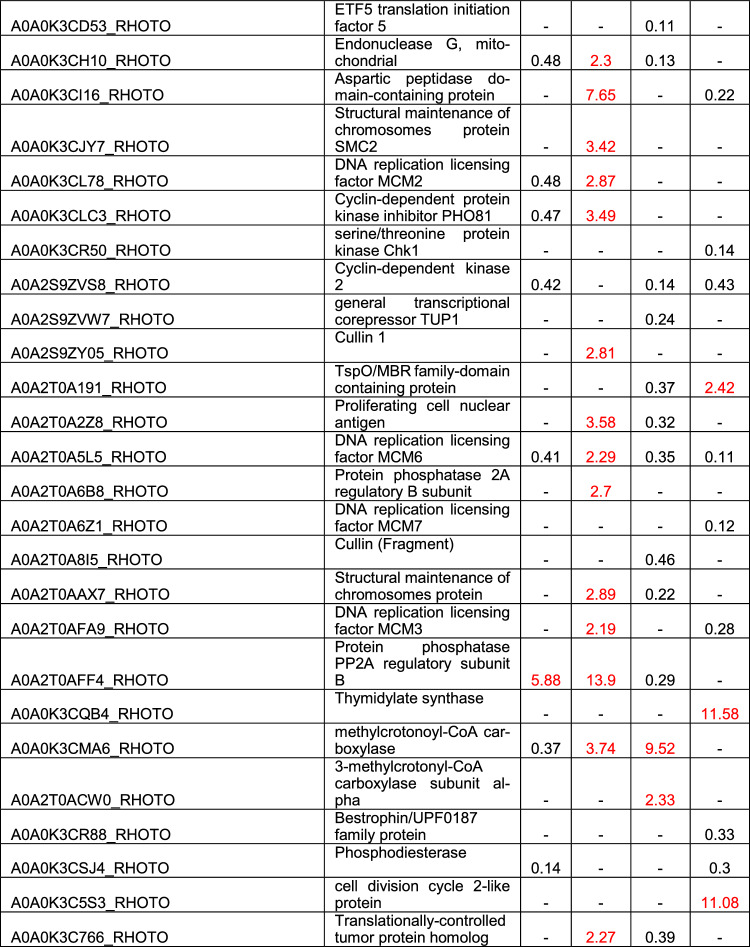
Discussed differentially regulated proteins discussed above involved in apoptosis, cell cycle regulation and cell proliferation

Proteins depicted with a fold change of 2 and higher are upregulated (marked in red), while proteins with a fold change of 0.5 and lower are downregulated. Coditions in which no significant change was observed are marked by “- “. Fold change and significances of ≥ 2 were used as cut-off

In YPD, significantly more proteins were affected by rapamycin treatment, with similar numbers of proteins identified at 48 h and 120 h (15 and 19, respectively). At 48 h, bestrophin, a protein connected to cell proliferation in cancer [27, 28] was highly upregulated (30.74-fold) and several other proteins impacting cell cycle progression and cell proliferation were also upregulated to various levels. Among them, DNA replication licensing factors MCM2, MCM6 and MCM7, 2 cyclin-dependent protein kinases, as well as a translationally-controlled tumor protein (TCTP) homolog [24, 29–32]. The protein citron rho-interacting kinase, previously correlated with reduced cell proliferation and cell survival when knocked down or silenced, was found upregulated 4.37 -fold in IFO0559 YPD samples at 48 h [33–35]. A phosphodiesterase was identified with 2.65-fold upregulation. Phosphodiesterases have long been associated with cancer and uncontrolled cell proliferation. In fact, several works reviewed phosphodiesterase inhibitors as anti-cancer drugs due to phosphodiesterase oncogene activity [36, 37]. Interestingly, HIG1 a protein shown to inhibit cell proliferation and increase apoptotic activity, when overexpressed in colon adenocarcinoma cells [38], was significantly downregulated (0.15-fold).

At 120 h, expression levels of the identified phopshodiesterase dramatically increased to 28-fold. Three other proteins highly expressed compared to untreated samples comprise cathepsin D, the E3 ubiquitin-protein ligase ring box protein 1 (RBX1) and a cell division control protein. RBX1 was shown to hold significant prognostic value in certain types of cancer, with high expression correlating to poor prognosis [39–41]. Among other proteins, DNA replication licensing factor MCM7, methylcrotonoyl-CoA carboxylase, thymidylate synthase, adenylate cyclase, mitogen-activated protein kinase, serine/threonine protein kinase Chk1 and cell division control protein cdc28, also known as cdc2 and cyclin-dependent kinase 1 [42], were all upregulated 2- to fivefold. All of which were previously connected to increased cell proliferation when dysregulated [24, 43–50].

A cyclin-dependent protein kinase identified among the upregulated proteins in IFO0559 YPD samples at 48 h, was downregulated 0.46-fold at 120 h of cultivation.

Another protein differentially expressed among the IFO0559 samples is the casein kinase II protein or rather its alpha and beta subunits, which were identified within YNB and YPD samples. Most significantly, both subunits were upregulated at 48 h in YPD samples (alpha 2.29-fold, beta 6.49-fold), while the alpha subunit was also identified in YNB at 120 h (3.44-fold) and the beta subunit was also upregulated in YPD at 120 h (3.03-fold).

In case of IFO0880, 42 proteins of the same categories were significantly impacted by rapamycin treatment (Table 3, S7&S8). In contrast to IFO0559, significantly more proteins were downregulated in IFO880. When cultured in YNB, 7 proteins were downregulated at 48 h, and 2 proteins upregulated. Among those, Protein phosphatase PP2A regulatory subunit B (5.88-fold), adenylate cyclase (2.05-fold), endonuclease G (0.48-fold), DNA replication licensing factor MCM2 and MCM6 (0.48-fold and 0.41-fold), cyclin-dependent kinase 2 (0.42-fold), which is associated with several types of cancer when overactive [29], and Methylcrotonoyl-CoA carboxylase (0.37-fold). Interestingly, methylcrotonoyl-CoA carboxylase and Protein phosphatase PP2A regulatory subunit B were inversely regulated in YPD samples at the same time point with 9.52-fold upregulation and 0.29 downregulation, respectively. Proteins upregulated at 48 h in IFO0880 YPD samples are ribonuclease T(2) and a serine/threonine-protein phosphatase 2A catalytic subunit. However, significantly more proteins were downregulated including 2 HIG1 domain-containing proteins, endonuclease G, DNA replicating licensing factor MCM6, general transcriptional corepressor TUP1 and proliferating cell nuclear antigen, a well-known prognostic marker and oncogene [51, 52].

At 120 h, 16 proteins were differentially regulated in YPD samples with only 5 proteins upregulated. Spindle Assembly checkpoint component MAD1 shows the highest upregulation holding significant importance in checkpoint control during mitosis [53]. Other proteins significantly upregulated are the thymidylate synthase, a cell division cycle 2-like protein, a Cytochrome c-like domain-containing protein and a TspO/MBR family-domain containing protein, with the first two exhibiting more than 11-fold increase compared to untreated samples. Among the downregulated proteins a HIG1-domain-containing protein, DNA replicating licencing factors MCM3, MCM6 and MCM7, cyclin-dependent kinase 2, mitogen-activated protein kinase, serine/threonine protein kinase Chk1 and cathepsin D, with HIG1, MCM6 and MCM7 exhibiting the highest differential regulation (0.06-fold, 0.11-fold and 0.12-fold, respectively). However, the most severe downregulation was identified for E3 ubiquitin-protein ligase RBX1. Interestingly, most of these proteins were upregulated in IFO0559 samples.

For YNB samples at 120 h, several of those same proteins were identified with differential regulation. Among those, cathepsin D, methylcrotonoyl-coA carboxylase and DNA replication licensing factor MCM6 which were upregulated in YNB samples (7.65-fold, 3.74-fold and 2.29, respectively). Another protein upregulated in YNB samples at 120 h, which was downregulated in YPD samples was a Ras-family domain containing protein (2.24-fold in YNB; 0.38-fold in YPD).

#### Ribosomes and translation

Rapamycin is known for its effect on ribosome biogenesis [8, 11]. In this study, 28 proteins involved in ribosome biogenesis were differentially regulated in IFO0559 cultures upon rapamycin treatment (S7&S8). Notably, most ribosome biogenesis proteins (21) were identified in YNB samples, most at 120 h (19). Almost all differential expressed ribosomal proteins were identified at 120 h (76 of 80) (S7 & S8). In YPD cultures of IFO0559 at 48 h, only two ribosomal proteins and two proteins involved in ribosome biogenesis, casein kinase II subunits alpha and beta (2.29-fold and 6.49-fold upregulation), were differentially regulated. Casein kinase II also plays a crucial role in cell cycle regulation and was found overexpressed in different types of cancer [54].

In IFO0880 22 distinct differentially expressed ribosome biogenesis proteins were identified. 15 proteins were identified in YNB samples and 13 in YPD samples, 12 at 48 h and 16 at 120 h. In total 79 ribosomal proteins were differentially expressed in IFO0880, 66 of which were identified at 120 h of cultivation, while only 21 were identified at 48 h of cultivation. In YNB samples 24 proteins were differentially expressed, while 71 were identified for YPD cultured samples. Significantly contrasting IFO0559, 41 ribosomal proteins and 8 ribosome biogenesis proteins were differentially expressed in YPD samples of IFO0880 at 48 h most of those (38 and 8) significantly downregulated (S7&S8).

In regard to proteins involved in translation, 19 proteins were differentially expressed in rapamycin treated IFO0559 samples and 23 proteins in IFO0880. However, at 48 h only 1 protein of this group was identified in IFO0559 in YPD, being the translationally-controlled tumor protein (TCTP) homolog with 2.1-fold upregulation. TCTP is known to be involved in cell growth and cell division and tends to accumulate to higher levels in tumors than in normal tissue [24, 29–32]. Interestingly, the same protein is downregulated in YPD samples of IFO0880 at 48 h (0.39-fold). Furthermore, TCTP is upregulated in both haplotypes in YNB at 120 h and in IFO0559 it is also upregulated in YNB at 48 h, showing a noticeable difference to the IFO0880 haplotype.

In general, most of the identified proteins are upregulated in IFO0559 samples with exception of proteins found in YPD samples at 120 h, where IFO0559 already entered the stationary phase, whereas in IFO0880, most proteins are downregulated upon rapamycin treatment, except in cultures grown in YNB, which predominantly exhibit upregulation of translational proteins. YPD samples of IFO0880 at 120 h, show slightly more upregulated proteins than those at 48 h.

#### Amino acid metabolism

Amino acid sustenance is critical for TOR pathway regulation [8, 18]. Therefore, proteins involved in amino acid biosynthesis were analyzed. Several proteins were significantly upregulated, especially at 120 h (38 and 35 for IFO0559 YNB and IFO0559 YPD, respectively; 70 and 16 for IFO0880 YNB and IFO0880 YPD, respectively) (Table 4, S7 & S8). Notably, only 5 proteins exhibit downregulation in IFO0559 at 120 h, all of which were identified in YPD samples. In IFO0880 YPD samples, the number of downregulated proteins is significantly higher (27). For YNB samples of IFO0880, only 4 downregulated proteins were identified at 120 h.

**Table 4:**
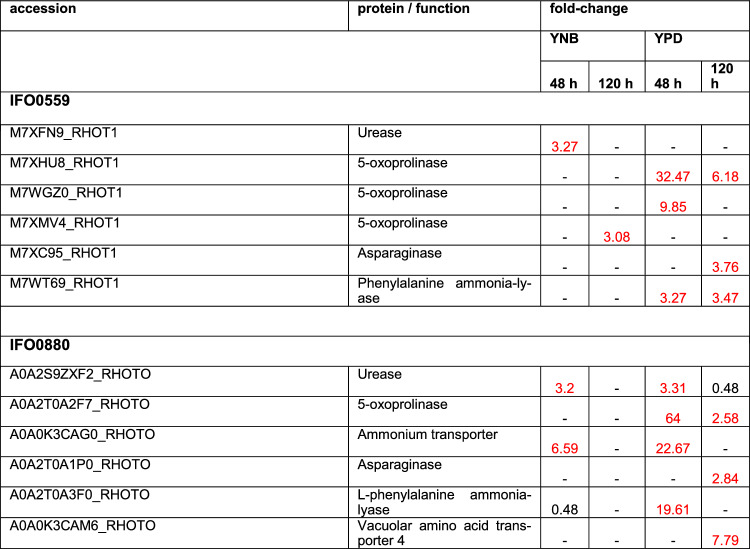
Discussed differentially regulated proteins impacting free amino acid levels

Proteins depicted with a fold change of 2 and higher are upregulated (marked in red), while proteins with a fold change of 0.5 and lower are downregulated. Coditions in which no significant change was observed are marked by “- “. Fold change and significances of ≥ 2 were used as cut-off

At 48 h, 29 proteins of amino acid metabolism were differentially regulated in IFO0559 upon rapamycin treatment, 19 of which were only found in YPD samples with 16 of those exhibiting upregulation. Interestingly, significantly more proteins were identified within IFO0880 samples at 48 h (85 distinct proteins), most of which exhibit significant downregulation, especially in YPD.

When limiting to amino acid biosynthesis proteins, proteins identified within YNB samples of IFO0880 are almost exclusively upregulated (44 of 47 at 120 h; 15 of 22 at 48 h), while YPD samples of IFO0880 exhibit predominantly downregulation (18 of 21 at 120 h; 28 of 35 at 48 h). In IFO0559 amino acid biosynthesis is mainly upregulated.

Among these proteins, 8 for IFO0559 and 16 for IFO0880, are involved in the biosynthesis of arginine (Table 4, S7&S8). Arginine was shown to increase cell proliferation and reduce apoptosis [55].While proteins involved in arginine biosynthesis identified in IFO0559 were almost exclusively upregulated, those found in YPD grown IFO0880 samples were almost exclusively observed in decreased abundance upon rapamycin treatment. In YNB cultures of IFO0880 little change was observed at 48 h, but samples exhibited upregulation of 6 arginine biosynthesis related proteins at 120 h.

Notably, 5-oxoprolinase was severely upregulated in samples grown in YPD medium for both haplotypes (Table 4). Some studies suggest, that 5-oxoprolin levels correlate with nutritional status and might be involved in amino acid uptake. Levels of 5-oxoprolinase were found to be dysregulated in several types of human cancer cells, however due to limited data, specific connections are still inconclusive [56, 57]. In IFO0559, a second 5-oxoprolinase was identified upregulated in YPD at 120 h and a third in YNB grown samples at 120 h. IFO0880 shows additional upregulation of an ammonium transporter potentially indicating ammonium accumulation for amino acid biosynthesis.

Furthermore, both IFO0559 and IFO0880 show significantly increased levels of phenylalanine-ammonia lyase at 48 h when cultured in YPD.

Asparaginase, an enzyme known as cytostatic, capable of inhibiting TOR mediated signaling resulting in ribosome biogenesis [58, 59], was upregulated in both haplotypes at 120 h when cultured in YPD (Table 4).

Another protein identified within the samples is the vacuolar amino acid transporter 4, which was 7.79-fold upregulated within the YPD samples of IFO0880 at 120 h of cultivation.

#### Autophagy, endocytosis, phagocytosis and proteolysis

We further analyzed differential regulation of autophagy, endocytosis, phagocytosis and proteolysis all of which are able to break down endogenous non-essential or imported substrates in order to improve nutritional status. This way, the cell is able to modulate free amino acid levels, as well as fatty acid or carotenoid profile independent of biosynthesis. Furthermore, these processes are of central importance in aging and neuro-degenerative diseases [60, 61]. In many organisms, autophagy is activated upon nutrient deprivation, which is mediated through the inactivation of TORC1. Therefore, rapamycin is known to induce autophagy in many of those cell types [62, 63].

For IFO0559 and IFO0880, 53 and 106 proteins involved in these processes were differentially regulated (S7 & S8). Interestingly, in YNB samples of IFO0559, the respective proteins were mostly upregulated, while in YPD samples of the same haplotype, notably more proteins were downregulated.

Proteins involved in autophagy were mainly downregulated, while proteins of the phagosome were primarily identified in YNB samples, most of which were upregulated. Lysosomal proteins and proteins involved in endocytosis were upregulated in both YPD and YNB samples.

In IFO0880, it is apparent, that proteins produced by YNB grown cultures at 120 h are almost exclusively upregulated, while in YPD, numbers of proteins upregulated and downregulated are similar. At 48 h, almost all proteins involved in these processes were downregulated in IFO0880. Furthermore, rapamycin treatment impacted significantly more proteins involved in autophagy in IFO0880 compared to IFO0559.

Notably, a protein upregulated in IFO0880 and IFO0559 samples, phospholipase D, was shown to confer rapamycin resistance in breast cancer [64, 65].

#### Fatty acid and carotenoid metabolism

As a result of the changes observed in carotenoid and fatty acid content upon rapamycin treatment, the proteomics data was mined for changes in the respective metabolic pathways. Carotenoids are synthesized from isoprenoid building blocks. In *R. toruloides* and other carotenogenic yeast, isoprenoids are built via the mevalonate pathway. Of those enzymes involved, the 3-hydroxy-3-methyl-glutaryl-coenzyme A (HMG-CoA) reductase is a well-known bottleneck [66, 67]. Within the proteomics data, HMG-CoA reductase was upregulated in IFO0559 in YPD at 48 h (7.36-fold) (Table 5), correlating with the significant increase in carotenoid accumulation observed. However, IFO0559 HMG-CoA reductase levels were additionally increased in YNB samples at 120 h. In IFO0880, the enzyme was downregulated at both time points when cultured in YPD medium (0.36-fold and 0.45-fold at 48 h and 120 h, respectively). A previous study by Seo et al. also established downregulation of HMG-CoA reductase (mRNA) upon rapamycin treatment in two colorectal cancer cell lines [68].

**Table 5:**
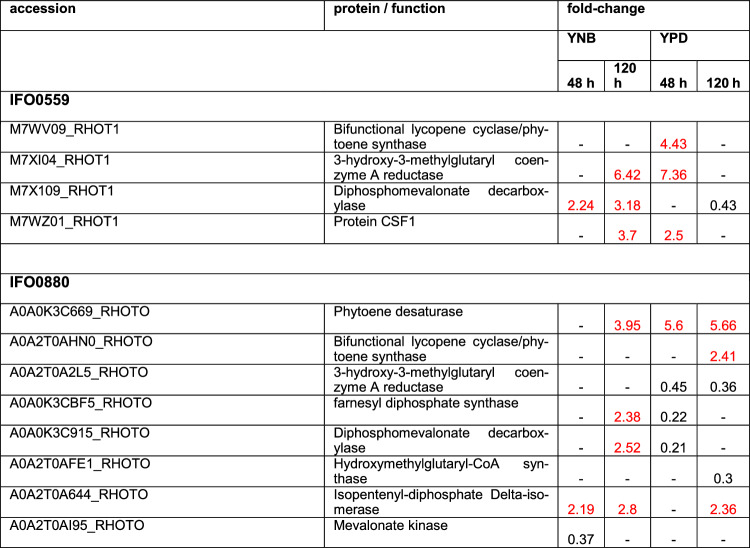
Discussed differentially regulated proteins involved in carotenoid biosynthesis

Proteins depicted with a fold change of 2 and higher are upregulated (marked in red), while proteins with a fold change of 0.5 and lower are downregulated. Coditions in which no significant change was observed are marked by “- “. Fold change and significances of ≥ 2 were used as cut-off

Other enzymes of the mevalonate pathway, such as diphosphomevalonate decarboxylase, HMG-CoA synthase, mevalonate kinase and isopentenyl-diphosphate delta-isomerase were almost exclusively downregulated when identified in YPD samples with most of these enzymes only identified in YPD samples of IFO0880, not in those of IFO0559. In analyzed samples of YNB grown cultures, the same enzymes were mostly upregulated or not significantly altered, with only the mevalonate kinase downregulated in IFO0880 at 48 h.

Of the carotenoid biosynthesis pathways itself, few proteins involved were identified. Most notably, the bifunctional lycopene cyclase/phytoene synthase was significantly upregulated (4.43-fold) in IFO0559 grown in YPD at 48 h and in IFO0880 when cultured in YPD for 120 h (2.41-fold), correlating with a significant increase of carotenoids in both haplotypes. In IFO0880, the phytoene desaturase was also upregulated 3.95-fold and 5.66 -fold in YNB and YPD at 120 h, respectively and 5.6-fold in YPD at 48 h.

Concerning fatty acid metabolism, a diverse set of proteins was differentially regulated upon rapamycin treatment. Proteins identified were involved in fatty acid degradation, fatty acid biosynthesis and fatty acid elongation. Interestingly, for IFO0559, most proteins involved in fatty acid biosynthesis were observed in YNB samples, while in IFO0880, similar numbers of proteins were differentially regulated across YNB and YPD samples. However, proteins specific to biosynthesis of unsaturated fatty acids were predominantly found in YPD samples.

In both haplotypes, most differentially regulated proteins involved in fatty acid degradation were found in YPD samples, which were predominantly upregulated in IFO0559 samples (8 upregulated, 2 downregulated), while proteins in IFO0880 YPD samples were up- and downregulated in similar amounts (6 upregulated, 7 downregulated).

Furthermore, CSF1, which is hypothesized to import lipids as substrate for ER-localized lipases, was upregulated 2.5-fold in IFO0559 YPD samples at 48 h, correlating with an early increase in fatty acid content, although not significant. Through ER lipase activity, IFO0559 could generate free fatty acids from those imported lipids [69, 70].

## Discussion

The known involvement of TOR signaling in many human diseases, especially cancer and aging-related diseases, make it an intensely studied subject [8, 14, 16]. To date, *S. cerevisae* serves as an important model for TOR signaling in mammalian systems. In fact, the TOR pathway was identified in *S. cerevisae*. However, *S. cerevisae* includes two different TOR kinases (TORC1 and TORC2), while mammalian cells express only produce one TOR kinase as member of both complexes [8, 9]. Since its discovery, the TOR pathway was also identified in other yeasts including the oleaginous yeast *R. toruloides*, which, equivalent to mammalian cells, only comprises one TOR kinase involved in both TOR complexes [12, 22]. In addition, *R. toruloides* can accumulate high amounts of lipids in form of lipid bodies or lipid droplets, organelles, gradually recognized as another prominent constituent of several human malignancies, essentially acting as an independent organelle inside the cell. Lipid droplets are linked to cell proliferation, cell survival, invasion, metastasis, as well as chemotherapy resistance [71]. Therefore, models such as *R. toruloides*, capable of naturally producing high amounts of cellular lipids, are of high interest for future studies focusing on lipid droplets and their function in cancer [1–3]. Rapamycin, an inhibitor of TOR signaling, tested in several studies as anti-cancer drug, was also associated with changes in lipid content [13, 72]. Especially in this context, studies on a new model, naturally apt to accumulate high amounts of lipids and pigments help in generating new insights.

The differential effects of the rapamycin response in *R. toruloides* are elaborated below.

### Influence of rapamycin on growth, catorenoid content, lipid content and on lipid profile

TOR signaling affects proliferation, apoptosis and energy metabolism [8, 14, 22]. Therefore, OD and biomass accumulation were monitored during the cultivation period. Treatment of YNB grown cultures only resulted in slight changes compared to untreated samples. As TOR signaling is affected by nutritional availability, TOR signaling is likely already inhibited by nutrient deprivation in minimal medium, especially amino acid availability. Hence, our observation of YNB grown cultures supplemented with rapamycin behaving highly similar to their respective controls, which is congruent with data collected in other oleaginous yeast [22].

Interestingly, the two different haplotypes used in this study exhibited pronounced differences in response to rapamycin when cultured in full medium. While IFO0559 almost seemed unaffected at first with only small delay in growth within the first 24 h, IFO0880 exhibited severely prolonged inhibition of growth between 20 and 80 h of cultivation. It stands to reason, that this effect can be in part contributed to the already increased lag-phase observed in IFO0880, in which adaption takes significantly longer. Therefore, adaption to rapamycin might also be significantly prolonged. Similar results were shown previously, when subjected to copper I treatment, in which a significantly prolonged adaption phase of IFO0880 was observed [3]. Additionally, IFO0880 might be more susceptible to inhibition by externally applied components as it could also be reasoned, that concentration of both Cu(I) and rapamycin decrease over time while cell count increases and therefore IFO0880 might slowly overcome inhibiting effects of the substance while IFO0559 might be more tolerant.

In previous studies, an increase in biomass of *S. cerevisae* was documented after long-term exposure to low doses of rapamycin [18] and treatment with rapamycin at a concentration of 5 µM increased biomass and lipid accumulation of *C. oleaginosus* in full medium [22].

Similar to Bracharz et al., rapamycin treatment resulted in an increase in lipids, when grown in YPD, potentially acting as additional organelle in avoidance of apoptosis similar to cancer cells. Interestingly, carotenoid levels were also increased upon treatment with rapamycin when cultured in full medium. Carotenoids, as well as other antioxidants, are widely studied in cancer and other aging-related diseases as they impact cell proliferation, apoptosis and other processes in eukaryotic cells including human cancer cells [71, 73–80]. Therefore, holistic studies and new models such as *R. toruloides* evaluating the interdependencies of TOR signaling, lipid accumulation and antioxidant accumulation are urgently needed. Notably, maximum carotenoid accumulation was significantly higher in IFO0559 than in IFO0880, while the highest lipid accumulation was observed in IFO0880 cultures. The differences between the two haplotypes in accumulation of carotenoids and lipids, might also affect behavior upon rapamycin treatment as both lipids in form of lipid droplets, as well as carotenoid content can impact cell survival and cell proliferation. Carotenoids, such as lycopene, ß-carotene and astaxanthin, were shown to reduce cell proliferation and induce apoptosis in different eukaryotic cells including cancer cells [73–77]. In contrast, lipid droplets were identified as important organelles in cancer, acting as energy reservoir, with increased lipid droplet density in many different human malignancies, including breast cancer, prostate cancer and colorectal cancer [71]. Interestingly, *R. toruloides* IFO0559, producing significantly more carotenoids and less lipids than IFO0880, adapts more quickly to rapamycin, while IFO0880 growth stagnates. One reason might be, that IFO0559 has developed mechanisms to cope with these antiproliferative and pro-apoptotic effects due to its naturally high carotenoid content, therefore showing higher resistance to drugs and substances inducing similar effects.

To further understand adaptation mechanisms induced by rapamycin treatment, the data was complemented by quantitative proteomics, which was performed during early and late stages of cultivation (48 h and 120 h) for both haplotypes in both full and minimal medium.

Proteomics identified a significant increase in protein levels of the bifunctional lycopene cyclase/phytoene synthase (4.43-fold) of IFO0559 at 48 h in YPD medium, correlating with the significant increase in carotenoid levels. Phytoene synthase is one of the rate limiting steps in many carotenoid biosynthesis pathways [81]. It catalyzes the transformation from geranylgeranyl pyrophosphate to phytoene. As bifunctional lycopene cyclase/phytoene synthase, the protein also directs metabolic flux away from lycopene to β-carotene [66, 82]. In a previous study, it was shown that β-carotene has less influence on cell viability than lycopene. It is therefore conceivable, that this already represents an initial adaptation. In accordance to our findings in *R. toruloides*, the microalgae *Chlamydomonas reinhardtii* was shown to produce increased amounts of carotenoids (Neoxanthin, Violaxanthin, Anteraxanthin and Lutein) when treated with rapamycin [83], further illustrating the effect of TOR signaling on carotenoid biosynthesis.

The main bottleneck of the mevalonate pathway, HMG-CoA reductase, essential for isoprenoid precursor production was also upregulated, representing the main precursors for carotenoid biosynthesis [66–68].

At 120 h, neither members of the mevalonate pathway, nor proteins involved in carotenoid biosynthesis were differentially regulated in YPD samples of IFO0559. Notably, carotenoid levels for rapamycin treated samples grown in minimal media decreased at both time points compared to untreated samples.

Interestingly, in IFO0880 grown in YPD medium, proteins of the mevalonate pathway, including the bottleneck HMG-CoA reductase, were observed in reduced abundance at both time points, while two proteins involved in carotenoid biosynthesis, a bifunctional lycopene cyclase/phytoene synthase and a phytoene desaturase, as well as the protein isopentyl-diphosphate delta-isomerase, which helps in directing isoprenoid precursors into pathways, such as carotenoid biosynthesis [66], exhibited increased protein levels in YPD samples, indicating transformation of available precursors instead of de-novo synthesis, while IFO0559 samples indicate increase de-novo synthesis. This further correlates with previous studies, in which rapamycin treatment led to a decrease in HMG-CoA reductase mRNA levels in other cell types [68] and indicates another difference between haplotypes.

In case of fatty acid and lipid metabolism, proteomics revealed little impact of rapamycin treatment on IFO0559 samples grown in YPD for 48 h with 2 proteins decreased in abundance and 1 protein exhibiting increase levels, matching with the little change observed in fatty acid composition and overall lipid content. At 120 h, however, rapamycin treatment resulted in 12 proteins of fatty acid metabolism exhibiting significantly increased protein levels, most of which were matched to fatty acid degradation. Nonetheless, minor changes were observed in fatty acid composition. Interestingly, overall fatty acid content was increased in the respective samples.

Similarly, in YNB, few proteins involved in lipid metabolism were identified. Numbers of proteins involved in fatty acid degradation and fatty acid biosynthesis were similar, with degradation proteins slightly more abundant. Interestingly, CSF1, a protein hypothesized to import lipids from the plasma membrane [69, 70] was upregulated in IFO0559 in YNB at 120 h and in YPD at 48 h.

In case of IFO0880, significantly more proteins involved in fatty acid metabolism were identified, especially in samples cultivated for 48 h in YPD, in which rapamycin treatment had significant impact on the abundance of 12 proteins. Most proteins downregulated were involved in fatty acid degradation, correlating with the observed increase in fatty acid content. As rapamycin shows limited effect in minimal media, fatty acid content and composition as well as protein levels of enzymes involved in fatty acid metabolism were little impacted by in IFO0880 cultures grown in YNB.

When compared to previous studies performed on the proteomic changes during lipid droplet formation in *R. toruloides* induced by phosphate limitation, some of those proteins were found to be differentially regulated in this study as well [12, 84–86]. While phosphate limitation led to increased triacylglycerol biosynthesis, Wang et al. also observed a decrease in ribosome biogenesis, which is congruent with rapamycin activity. Interestingly, phosphate limitation did not seem to affect lipid degradation. In contrast, our proteomic analysis indicates involvement of rapamycin signaling in fatty acid degradation, recycling and import rather than just synthesis which could contribute to the higher impact on lipid levels when grown in rich media like YPD.

### Proteomic adaptation of processes regulating cell growth and survival

Growth and proteomic evaluation revealed a significantly higher impact of rapamycin treatment on IFO0880 than on IFO0559. Furthermore, rapamycin treatment exhibited significantly higher impact on YPD grown IFO0880 during the first 48 h of cultivation, than on samples cultured in YNB, while effects were more similar for IFO0559 cultures in both media.

#### TOR signaling

Among the proteins differentially regulated, several proteins of the TOR pathway were identified including the target of rapamycin itself, a serine/threonine kinase, which was differentially regulated in both haplotypes at 120 h when grown in YPD. Rapamycin binds to the protein FK506 Binding Protein 12 (FKBP12), the resulting complex then binds to TORC1 resulting in its inhibition by prevention of TORC1 substrate binding [8, 13]. TOR kinase protein levels were upregulated in IFO0880 YPD samples at 120 h, which could represent a mechanism of adaptation to rapamycin inhibition, as overexpression of the target of rapamycin itself might restore TOR signaling activity. Translation initiation factor eIF-4E, an important downstream target of TOR signaling, exhibits decreased protein levels at 48 h of cultivation in IFO0880 YPD cultures upon treatment with rapamycin, potentially through degradation, as TOR signaling is significantly impaired and proteolysis and autophagy are significantly impacted by TOR inhibition. However, at 120 h, with increased levels of TOR kinase expressed, eIF-4E is no longer differentially regulated. Notably, no differential regulation of eIF-4E was identified in IFO0559 YPD samples at 48 h. Deregulation of eIF-4E, e.g. by deregulation of eIF4E-binding protein 1, is known to cause rapamycin resistance in cancer [87–89]. Indicating another aspect of reduced sensitivity of IFO0559 to rapamycin.

At 120 h, YPD samples of IFO0559 exhibit reduced protein levels of TOR kinase and eIF-4E. This correlates with the collected growth data, as IFO0559 enters the stationary phase at around 90 h of cultivation.

No significant difference in TOR kinase levels was detected for YNB samples of either haplotype, as nutritional deprivation likely triggered similar effects [8, 72].

Interestingly, several v-type ATPases were differentially expressed in both haplotypes, which are responsible for lowering pH in endosomes and more specifically lysosomes [19, 21]. v-type ATPases are speculated to play a role in amino acid sensing, an important regulator of TOR activity. As they lower the pH in the lysosomes, they activate hydrolyzing enzymes involved in proteolysis, which can subsequently increase free amino acid levels by degradation of imported or endogenous proteins. Finally, free amino acids can elevate TORC1 activity [19, 21], potentially counteracting rapamycin treatment.

In fact, increase of intracellular free amino acids was shown as significant part of adaptation to prolonged rapamycin exposure in *S. cerevisae* [18]. Especially in IFO0880, v-type ATPase subunits were overexpressed in YNB samples at 120 h and to lesser extend at 48 h and in YPD samples at 120 h. In YPD at 48 h, IFO0880 exhibited significant downregulation of 2 v-type ATPase subunits correlating with severe growth inhibition.

Similarly, the protein transporter SEC13, a member of the GATOR2 complex, involved in amino acid sensing and subsequent activation of TORC1 [20], was upregulated in YNB samples of both haplotypes at 120 h. However, in IFO0880 samples grown in YPD at 48 h, SEC13 was significantly less abundant.

#### Apoptosis, cell cycle, cell proliferation

To further explain the significant difference rapamycin treatment had on the growth of the two haplotypes in full medium, expression levels of proteins involved in apoptosis, cell cycle and cell proliferation were analyzed.

Interestingly, IFO0559 samples, exhibited increased expression levels of several DNA replication licensing factors (MCM2, MCM6 and MCM7), despite rapamycin reportedly inhibiting MCM expression in several cell types [90], while the rapamycin-sensitive IFO0880 haplotype, exhibited reduced expression levels of MCMs upon rapamycin treatment in YPD. MCMs were previously correlated with cancer and unregulated cell proliferation [24, 25]. Furthermore, other proteins previously discussed in context of oncogenic potential were upregulated during early cultivation of IFO0559 in YPD, including bestrophin, citron rho-interacting kinase and CDK9 likely facilitating the rapid adaptation of IFO0559 samples. Bestrophins overexpression was strongly correlated to uncontrolled cell proliferation, especially, bestrophin 1 is discussed in this context [27, 28]. The citron rho-interacting kinase was shown to significantly reduce cell proliferation and cell survival when knocked out and CDK9 represents a key factor identified in various cancer diseases [33–35, 91]. Furthermore, a phosphodiesterase was upregulated specifically in IFO0559, while no direct analog was identified in IFO0880. Phosphodiesterases represent a much-researched target for cancer therapy. Notably, even in stationary phase at 120 h, prognostic markers for certain types of cancer correlated with poor prognosis were found upregulated in IFO0559 samples, while the same proteins were downregulated in IFO0880. Among those, RBX1 exhibited significant upregulation of 12.11-fold in IFO0559 at 120 h, and significantly reduced protein levels of only 0.05-fold in IFO0880. A second protein with increased expression in IFO0559, TCTP, was upregulated in YPD samples at 48 h, while IFO0880 samples exhibited downregulation of TCTP.

Casein kinase II, identified in increased abundance in IFO0559 samples, especially at 48 h in YPD, plays a crucial role in cell cycle regulation and cell proliferation. These enzymes are overexpressed in many different types of cancer [54]. Furthermore, casein kinase II was demonstrated to phosphorylate some members of FK506-binding proteins, demonstrating increased nuclear localization of FKBP25 upon phosphorylation. FKBP12, another member of this family is crucial for rapamycin mediated inhibition of TOR, as a dimer of FKBP12 and rapamycin initiates TOR1 inhibition. Potentially, casein kinase II overexpression could have similar effects on FKBP12 as it was shown for FKBP25, hence reducing FKBP12 levels in the cytoplasm [92]. FKBP12-rapamycin binding, takes place at the cytoplasm [93]. Therefore, reduced levels of cytoplasmic FKBP12, might lead to IFO0559s decreased rapamycin susceptibility.

Several changes were found consistent across all samples including the downregulation of a HIG1-domain containing protein. HIG1 was shown to inhibit cell proliferation and increase apoptotic activity in colon adenocarcinoma cells [38]. Downregulation of these proteins might therefore represent a common rapid adaption strategy that helps the cells in the evasion of apoptosis and in overcoming the anti-proliferatory effects of rapamycin. Furthermore, overexpression of the methylcrotonoyl-CoA carboxylase, a putative pro-proliferative protein, was observed across samples. Methylcrotonoyl-CoA carboxylase 2 overexpression is known as unfavorable prognostic marker in breast cancer, as it is involved in erratic cell proliferation [43, 44]. Thymidylate synthase, another protein correlated to increased cell proliferation was only found at 120 h of cultivation in both haplotypes [45].

At 120 h, reaching the stationary phase, IFO0559 and IFO0880 both exhibited increased levels of proteins involved in apoptosis. In IFO0559 YPD samples, levels of cathepsin D were increased by 19.39-fold [94]. Additionally, protein levels of the serine/threonine protein checkpoint kinase Chk1 correlated with G1 arrest were significantly increased [47]. In IFO0880, MAD1, a protein correlated to cell growth arrest, was significantly upregulated by 53.39-fold [53]. Additionally, IFO0880 exhibited increased protein abundance of endonuclease G, translocator protein TspO and cytochrome c. Tsp0 is involved in the release of cytochrome c from the mitochondria into the cytoplasm, which is an early event in apoptosis induced cell death [94–96]. During apoptosis, the mitochondrial endonuclease G is also released from the mitochondria into the cytoplasm acting as an apoptotic DNase. In previous studies, it was correlated with cell death of non-invasive human breast cancer cells [97, 98].

#### Ribosomes and translation

Several studies already established TOR signaling and hence rapamycin treatment to affect ribosome biogenesis and by extent ribosomal protein levels themselves [8, 11]. In this work, the effect of rapamycin on proteins involved in ribosome biogenesis, as well as on ribosomal proteins was evident. Although early samples of IFO0559 grown in YPD were little affected, many proteins were significantly downregulated in IFO0880, correlating with the distinct differences in growth of both haplotypes at early stages of cultivation and their different sensitivity to rapamycin. Similar effects were identified for translation initiation factors and other proteins involved in translation.

#### Amino acid metabolism

For model organism *S. cereviase*, it was shown, that adaptation to rapamycin includes upregulation of intracellular free amino acid levels, mainly by differential regulation of amino acid biosynthesis [18]. Furthermore, it was demonstrated, that specifically arginine seems to increase cell proliferation while reducing apoptosis [55].

In this study, both haplotypes show significant changes in amino acid metabolism upon rapamycin treatment. Although significantly more proteins involved in amino acid metabolism where differentially expressed in IFO0880 samples upon rapamycin treatment, amino acid biosynthesis was upregulated in all IFO0559 samples, while in IFO0880, upregulation appears predominantly in samples cultivated in YNB medium. The same is true for arginine biosynthesis related proteins. However, as amino acid supply in YPD medium is likely sufficient, expression of proteins involved in amino acid biosynthesis can likely decrease without limiting the intracellular supply of free amino acids at least during early cultivation. Therefore, import of amino acids was evaluated as another factor of adaptation. Notably, 5-oxoprolinase, as well as amino acid importers (SEC13, vacuolar amino acid transporter 4) and proteins involved in NH_3_ accumulation (ammonium transporter and phenylalanine-ammonia lyase) were also upregulated in both IFO0880 and IFO0559 when grown in YPD, with vacuolar amino acid transporter 4 and ammonium transporter only identified in IFO0880. 5-oxoprolinase plays a crucial role in the glutathione recycling pathway and its specific substrate, 5-oxoproline, is implicated in the mechanisms of nutrient sensing and amino acid assimilation, displaying an intricate relationship with glycine concentrations [57]. The enzymatic activity of 5-oxoprolinase may help in regulating 5-oxoproline levels and in turn may reflect a regulation of amino acid import and biosynthesis mediated by nutrient availability in the medium.

#### Autophagy, endocytosis, phagocytosis and proteolysis

Protein degradation represents a further mechanism by which cells can affect intracellular free amino acid levels. Processes such as autophagy are also key factors in cancer, aging and aging-related diseases in general [61, 99–103]. As rapamycin is known to activate autophagy and related processes in different cell types, it holds significant promises in the field of aging and neuro-degenerative diseases [16, 61–63]. In this study, many proteins involved in phagocytosis, endocytosis, autophagy, proteolysis and related processes were significantly impacted by rapamycin treatment. While cultures grown in YNB mostly exhibit upregulation of different proteins upon rapamycin treatment, fewer proteins were upregulated in YPD samples treated with rapamycin. Moreover, the impact of rapamycin-induced effects on autophagy-related proteins was found to be significantly less pronounced in IFO0559 than in IFO0880.

Interestingly, a protein increased in both haplotypes, phospholipase D, is known to confer rapamycin resistance in breast cancer [64, 65]. Phospholipase D was only identified in IFO0880 samples grown in YPD, not YNB, with significant upregulation at both time points. In IFO0559, phospholipase D was upregulated in YPD at both time points and in YNB at 120 h. It stands to reason, that increase in phospholipase D levels indicate one part of the adaption mechanisms of *R. toruloides* to rapamycin during cultivation period.

#### Conclusion

In this work, we presented a first model of *R. toruloides* allowing for the combinatorial analysis of lipid droplets as emerging hallmark of cancer together with pigment accumulation and a more mammalian like TOR pathway in the context of rapamycin treatment. By proteomic analysis we were able to identify adaption previously described in the context of human disease. Key proteins found in this study include altered expression of translation initiation factors such as eIF-4E which is known to drive oncogene translation, contributing to tumor formation and involvement in rapamycin resistance. Furthermore, expression V-type proton ATPases was significantly altered, which correlates with acidification of intracellular compartments, protein degradation, tumor invasiveness and drug resistance in cancer likely including rapamycin. MAP-kinases and cyclin-dependent kinases are crucial for cell cycle regulation, with dysregulation leading to uncontrolled proliferation commonly found in diverse cancer types. Further, proteins correlated with cell cycle progression and apoptosis such as cathepsin D, MCM proteins and casein kinase II, all correlated with different types of cancer, were significantly affected during adaption to rapamycin.

These parallels between *R. toruloides* and human biology, especially regarding the TOR pathway and associated proteins, show great promise of this yeast as a model. Particularly the existence of two haplotypes with significantly different responses to rapamycin treatment shed light on mechanisms likely involved in rapamycin resistance. However, significantly more in-depth analyses of *R. toruloides*, its behavior and its pathways will be necessary, to assemble a fully functional surrogate model for TOR signaling in aging-related diseases. We are convinced, that the use and comparison of both haplotypes can be of significant assistance in the evaluation of other metabolic changes and their connection to disease, where small changes in lipid content, carotenoid content or sensitivity to a drug are of utmost importance.

## Materials and methods

### Yeast strain and culture conditions

Seed-cultures of *R. toruloides* (IFO0559 & IFO0880) were cultivated in 100 mL baffled flasks filled with 20 mL of either yeast-nitrogen base medium (6.7 g L-1 Yeast-Nitrogen Base [without Amino Acids] Carl Roth GmbH & Co. KG (Karlsruhe, Germany), 40 g L-1 glucose) or yeast extract-peptone-dextrose (YPD) medium (10 g L-1 yeast extract, 20 g L-1 peptone, 40 g L-1 glucose).

For all experiments, 500 mL baffled flasks with Duran GL32 Membrane Vented Screw caps (DWK Life Science, Wertheim, Germany) filled with 125 mL YNB or YPD were inoculated to an OD of 0.2, followed by 120 h of cultivation in a rotary incubator (New Brunswick InnovaTM 44, Eppendorf, Hamburg, Germany) at 28 °C, 100 rpm. All cultivations were performed in biological triplicates.

For rapamycin treatment, 125 µL of a 5 mM Rapamycin in DMSO stock solution were added to a final concentration of 5 µM. In YNB, 0.03% ammonia were added to the media, as previous testing demonstrated a drastic increase in IFO0880 growth [3].

### Sample collection

Sampling for OD600nm measurement was performed twice a day. Following a 48-h cultivation period, a significant difference in turbidity was observed between IFO0559 and IFO0880, indicating substantial differences in growth patterns. Consequently, the 48-h cultivation period was selected for the initial sampling. Furthermore, a sample of the late culture was taken to check for further changes and adaptation, especially since IFO0880 was able to overcome the inhibitory effects of rapamycin and grow to biomasses higher than untreated samples after around 100 h.

10 mL of culture were sampled for DCW, lipids and carotenoids and another 10 mL for proteomics. Samples were centrifuged at 4000 g for 10 min and the supernatant was discarded. For YNB cultures, samples were processed without further treatment. For YPD samples, all samples were washed with 10 mL ddH2O before processing. After centrifugation, samples were temporarily stored at  – 80 °C.

### Growth analysis

Growth analysis was performed measuring optical density of the cultures at 600 nm (Nano Photometer NP80, IMPLEN, Munich, Germany). Polystyrene standard semi-micro cuvettes were filled with a sample volume of 1 mL.

For DCW determination, the previously collected pellets were dried by lyophilization ( – 80 °C, min. 72 h), followed by gravimetric measurement.

### Pigment extraction

Pigment extraction was performed as previously described [3]. On average, 8 mg of dry biomass were disrupted with glass beads (2 mm) and carotenoids were extracted with acetone, followed by absorbance measurement at 454 nm (Nano Photometer NP80, IMPLEN, Munich, Germany).

### Fatty acid profile

3 mg of dry biomass were sampled and analyzed via fatty acid methyl ester (FAME) analysis as described previously [3, 104]. In summary, conversion to fatty acid methyl esters was performed utilizing a MultiPurposeSampler MPS robotic (Gerstel, Linthicum Heights, US). The resulting compounds were then analyzed by gas chromatography (GC-2025 coupled to AOC-20i Auto injector and AOC-20 s Auto sampler, Shimadzu, Duisburg, Germany) using a flame ionization detector. Separation was achieved by a Zebron ZB-wax column (Phenomenex, Aschaffenburg, Germany).

As previous testing indicated the absence of C12:0, methyl laurate (C12; Restek GmbH, Bad Homburg, Germany) was used as internal standard. As external standard, Marine Oil FAME mix (20 components from C14:0 to C24:1; Restek GmbH, Bad Homburg, Germany) and FAME #12 mix (C13:0, C15:0, C17:0, C19:0, C21:0; Restek GmbH, Bad Homburg, Germany) were utilized.

### Proteomics

#### Proteom extraction and measurement

As previously shown [3], proteins were extracted using Protein Extraction Reagent Type 4 (Sigma-Aldrich, St. Louis, US) (1:3, v/v) and precipitated with 20% (v/v) trichloroacetic acid. After washing, protein pellets were resuspended in urea supplemented with DTT followed by in-gel digestion utilizing trypsin.

Peptide analysis was performed using a timsTOF Pro mass spectrometer equipped with a NanoElute LC System (Bruker Daltonik GmbH, Bremen, Germany) with an Aurora column (250 × 0.075 mm, 1.6 μm; Ion-Opticks, Hanover St., Australia).

#### Bioinformatics analysis

Peptide and protein identification was performed using PEAKS Studio software 10.6 (Bioinformatics Solutions Inc., Waterloo, ON, Canada) [105–107]. Databases for protein identification in IFO0880 and IFO0559 samples were obtained from Uniprot (IFO0559: https://www.uniprot.org/proteomes/UP000016926, 8138 proteins and IFO0880: https://www.uniprot.org/proteomes/UP000239560, 8475 proteins).

Parameter settings for peaks analysis include precursor mass 25 ppm, monoisotopic mass and fragment ion 0.05 Da, trypsin digestion, a maximum of two missed cleavages per peptide, FDR 1.0% and at least 1 unique peptide per protein.

For comparison between the different experimental conditions, PEAKSQ quantification tool was employed, with a set mass error tolerance of 20.0 ppm, ion mobility tolerance 0.05 Da and the retention time shift tolerance at 6 min (auto detect). For data evaluation, only proteins with significance and fold change ≥ 2 were considered.

Functional characterization was supported by KOALA (KEGG Orthology And Links Annotation, https://www.kegg.jp/blastkoala/).

## Supplementary Information

Below is the link to the electronic supplementary material.

Supplementary file1 (DOCX 1362 KB)

Supplementary file2 (XLSX 26 KB)

Supplementary file3 (XLSX 153 KB)

Supplementary file4 (XLSX 130 KB)

Supplementary file5 (XLSX 173 KB)

## Data Availability

All relevant data can be found within the paper, the supplementary material and on the PRIDE partner repository with the dataset identifier PXD05238. Supplementary data S1-S4 includes supplemental data on fatty acid profile, growth behavior and *R. toruloides* TOR kinase sequence analysis. Original data of OD and DCW is included in S5. Original data of Fatty acids and carotenoid analysis can be found in S6. The proteomics results of IFO0559 are given in S7 and proteomics results of IFO0880 in S8. Proteomics raw data can be accessed via PRIDE: Reviewer access details: 744. Unique link: https://www.ebi.ac.uk/pride/review-dataset/382b7417f480486991f183a53c8283bb 745. Project accession: PXD052386 746. Token: qfflVi890eDV 747. Alternatively, reviewers can access the dataset by logging into the PRIDE website using the following account details: 748. Username: reviewer_pxd052386@ebi.ac.uk 749. Password: qKmD2VWPsgJI.

## References

[1] Wen Z, Zhang S, Odoh CK, Jin M, Zhao ZK (2020) *Rhodosporidium toruloides* - A potential red yeast chassis for lipids and beyond. FEMS Yeast Res. 10.1093/femsyr/foaa038 32614407 10.1093/femsyr/foaa038 PMC7334043

[2] Zhao Y, Song B, Li J, Zhang J (2022) *Rhodotorula toruloides*: an ideal microbial cell factory to produce oleochemicals, carotenoids, and other products. World J Microbiol Biotechnol 38:13. 10.1007/s11274-021-03201-4 10.1007/s11274-021-03201-4 34873661

[3] Cavelius P, Engelhart-Straub S, Biewald A, Haack M, Awad D, Brueck T, Mehlmer N (2023) Adaptation of proteome and metabolism in different haplotypes of *Rhodosporidium toruloides* during Cu(I) and Cu(II) stress. Microorganisms 11:553. 10.3390/microorganisms11030553 36985127 10.3390/microorganisms11030553 PMC10056549

[4] Saini R, Hegde K, Osorio-Gonzalez CS, Brar SK, Vezina P (2020) Evaluating the potential of *Rhodosporidium toruloides*-1588 for high lipid production using undetoxified wood hydrolysate as a carbon source. Energies 13:5960. 10.3390/en13225960

[5] Zhang S, Skerker JM, Rutter CD, Maurer MJ, Arkin AP, Rao CV (2016) Engineering *Rhodosporidium toruloides* for increased lipid production. Biotech Bioeng 113:1056–1066. 10.1002/bit.25864 10.1002/bit.25864 26479039

[6] Hanson PK (2018) *Saccharomyces cerevisiae* : a unicellular model genetic organism of enduring importance. CP Essential Lab Tech 16:e21. 10.1002/cpet.21

[7] Karathia H, Vilaprinyo E, Sorribas A, Alves R (2011) Saccharomyces cerevisiae as a model organism: a comparative study. PLoS One 6:e16015. 10.1371/journal.pone.0016015 21311596 10.1371/journal.pone.0016015 PMC3032731

[8] Eltschinger S, Loewith R (2016) TOR complexes and the maintenance of cellular homeostasis. Trends Cell Biol 26:148–159. 10.1016/j.tcb.2015.10.003 26546292 10.1016/j.tcb.2015.10.003

[9] Loewith R, Jacinto E, Wullschleger S, Lorberg A, Crespo JL, Bonenfant D, Oppliger W, Jenoe P, Hall MN (2002) Two TOR complexes, only one of which is rapamycin sensitive, have distinct roles in cell growth control. Mol Cell 10:457–468. 10.1016/S1097-2765(02)00636-6 12408816 10.1016/s1097-2765(02)00636-6

[10] Wang T, Edgar BA (2010) TOR signaling and cell death. in the enzymes; Elsevier, Vol. 28, pp. 217–244 ISBN 978–0–12–381005–2

[11] Artoni F, Grützmacher N, Demetriades C (2023) Unbiased evaluation of rapamycin’s specificity as an mtor inhibitor. Aging Cell 22:e13888. 10.1111/acel.13888 37222020 10.1111/acel.13888 PMC10410055

[12] Zhu Z, Zhang S, Liu H, Shen H, Lin X, Yang F, Zhou YJ, Jin G, Ye M, Zou H et al (2012) A multi-omic map of the lipid-producing yeast *Rhodosporidium toruloides*. Nat Commun 3:1112. 10.1038/ncomms2112 23047670 10.1038/ncomms2112 PMC3493640

[13] Li J, Kim SG, Blenis J (2014) Rapamycin: one drug, many effects. Cell Metab 19:373–379. 10.1016/j.cmet.2014.01.001 24508508 10.1016/j.cmet.2014.01.001 PMC3972801

[14] Sabatini DM (2006) mTOR and cancer: insights into a complex relationship. Nat Rev Cancer 6:729–734. 10.1038/nrc1974 16915295 10.1038/nrc1974

[15] Zou Z, Tao T, Li H, Zhu X (2020) mTOR Signaling pathway and mTOR inhibitors in cancer: progress and challenges. Cell Biosci 10:31. 10.1186/s13578-020-00396-1 32175074 10.1186/s13578-020-00396-1 PMC7063815

[16] Lee DJW, Hodzic Kuerec A, Maier AB (2024) Targeting ageing with rapamycin and its derivatives in humans: a systematic review. Lancet Healthy Longevity 5:e152–e162. 10.1016/S2666-7568(23)00258-1 38310895 10.1016/S2666-7568(23)00258-1

[17] Xie J, Wang X, Proud CG (2016) mTOR inhibitors in cancer therapy. F1000Res 5:2078, 10.12688/f1000research.9207.1 10.12688/f1000research.9207.1 PMC5007757 27635236

[18] Dikicioglu D, Dereli Eke E, Eraslan S, Oliver SG, Kirdar B (2018) Saccharomyces cerevisiae adapted to grow in the presence of low-dose rapamycin exhibit altered amino acid metabolism. Cell Commun Signal 16:85. 10.1186/s12964-018-0298-y 30458881 10.1186/s12964-018-0298-y PMC6245637

[19] Zoncu R, Bar-Peled L, Efeyan A, Wang S, Sancak Y, Sabatini DM (2011) mTORC1 senses lysosomal amino acids through an inside-out mechanism that requires the vacuolar H ^+^ -ATPase. Science 334:678–683. 10.1126/science.1207056 22053050 10.1126/science.1207056 PMC3211112

[20] Valenstein ML, Rogala KB, Lalgudi PV, Brignole EJ, Gu X, Saxton RA, Chantranupong L, Kolibius J, Quast J-P, Sabatini DM (2022) Structure of the nutrient-sensing hub GATOR2. Nature 607:610–616. 10.1038/s41586-022-04939-z 35831510 10.1038/s41586-022-04939-z PMC9464592

[21] Stransky L, Cotter K, Forgac M (2016) The function of V-ATPases in cancer. Physiol Rev 96:1071–1091. 10.1152/physrev.00035.2015 27335445 10.1152/physrev.00035.2015 PMC4982037

[22] Bracharz F, Redai V, Bach K, Qoura F, Brück T (2017) The effects of TORC signal interference on lipogenesis in the oleaginous yeast Trichosporon Oleaginosus. BMC Biotechnol 17:27. 10.1186/s12896-017-0348-3 28270203 10.1186/s12896-017-0348-3 PMC5341401

[23] Jia L, Sun Y (2012) SCF E3 ubiquitin ligases as anticancer targets 10.2174/156800911794519734 PMC3323109 21247385

[24] Song S, Wang Y, Liu P (2022) DNA replication licensing factors: novel targets for cancer therapy via inhibiting the stemness of cancer cells. Int J Biol Sci 18:1211–1219. 10.7150/ijbs.67529 35173548 10.7150/ijbs.67529 PMC8771848

[25] Løkkegaard S, Elias D, Alves CL, Bennetzen MV, Lænkholm AV, Bak M, Gjerstorff MF, Johansen LE, Vever H, Bjerre C et al (2021) MCM3 upregulation confers endocrine resistance in breast cancer and is a predictive marker of diminished tamoxifen benefit. NPJ Breast Cancer 7:2. 10.1038/s41523-020-00210-8 33398005 10.1038/s41523-020-00210-8 PMC7782683

[26] Kozel C, Thompson B, Hustak S, Moore C, Nakashima A, Singh CR, Reid M, Cox C, Papadopoulos E, Luna RE et al (2016) Overexpression of eIF5 or its protein mimic 5MP perturbs eIF2 function and induces *ATF4* translation through delayed re-initiation. Nucleic Acids Res 44:8704–8713. 10.1093/nar/gkw559 27325740 10.1093/nar/gkw559 PMC5062967

[27] Kunzelmann K, Kongsuphol P, Aldehni F, Tian Y, Ousingsawat J, Warth R, Schreiber R (2009) Bestrophin and TMEM16—Ca2+ activated Cl− channels with different functions. Cell Calcium 46:233–241. 10.1016/j.ceca.2009.09.003 19783045 10.1016/j.ceca.2009.09.003

[28] Spitzner M, Martins JR, Soria RB, Ousingsawat J, Scheidt K, Schreiber R, Kunzelmann K (2008) Eag1 and Bestrophin 1 are up-regulated in fast-growing colonic cancer cells. J Biol Chem 283:7421–7428. 10.1074/jbc.M703758200 18222922 10.1074/jbc.M703758200

[29] Tadesse S, Caldon EC, Tilley W, Wang S (2019) Cyclin-dependent kinase 2 inhibitors in cancer therapy: an update. J Med Chem 62:4233–4251. 10.1021/acs.jmedchem.8b01469 30543440 10.1021/acs.jmedchem.8b01469

[30] Gao J, Ma Y, Yang G, Li G (2022) Translationally controlled tumor protein: the mediator promoting cancer invasion and migration and its potential clinical prospects. J Zhejiang Univ Sci B 23:642–654. 10.1631/jzus.B2100910 35953758 10.1631/jzus.B2100910 PMC9381325

[31] Bommer U-A, Thiele B-J (2004) The Translationally Controlled Tumour Protein (TCTP). Int J Biochem Cell Biol 36:379–385. 10.1016/S1357-2725(03)00213-9 14687915 10.1016/s1357-2725(03)00213-9

[32] Tuynder M, Fiucci G, Prieur S, Lespagnol A, Géant A, Beaucourt S, Duflaut D, Besse S, Susini L, Cavarelli J et al (2004) Translationally controlled tumor protein is a target of tumor reversion. Proc Natl Acad Sci USA 101:15364–15369. 10.1073/pnas.0406776101 15489264 10.1073/pnas.0406776101 PMC523462

[33] Sahin I, Kawano Y, Sklavenitis-Pistofidis R, Moschetta M, Mishima Y, Manier S, Sacco A, Carrasco R, Fonseca R, Roccaro AM et al (2019) Citron rho-interacting kinase silencing causes cytokinesis failure and reduces tumor growth in multiple myeloma. Blood Adv 3:995–1002. 10.1182/bloodadvances.2018028456 30940634 10.1182/bloodadvances.2018028456 PMC6457230

[34] Haiping C, Qi X, Dawei L, Qiang W (2019) Citron rho-interacting serine/threonine kinase knockdown suppresses prostate cancer cell proliferation and metastasis by blocking Hippo-YAP pathway 10.12122/j.issn.1673-4254.2019.03.01 PMC6765676 31068310

[35] Shou J, Yu C, Zhang D, Zhang Q (2020) Overexpression of citron Rho-interacting serine/threonine kinase associated with poor outcome in bladder cancer. J Cancer 11:4173–4180. 10.7150/jca.43435 32368300 10.7150/jca.43435 PMC7196275

[36] Peng T, Gong J, Jin Y, Zhou Y, Tong R, Wei X, Bai L, Shi J (2018) Inhibitors of phosphodiesterase as cancer therapeutics. Eur J Med Chem 150:742–756. 10.1016/j.ejmech.2018.03.046 29574203 10.1016/j.ejmech.2018.03.046

[37] Savai R, Pullamsetti SS, Banat G-A, Weissmann N, Ghofrani HA, Grimminger F, Schermuly RT (2010) Targeting cancer with phosphodiesterase inhibitors. Expert Opin Investig Drugs 19:117–131. 10.1517/13543780903485642 20001559 10.1517/13543780903485642

[38] Xu Z, Sun J, Mao Y, Chen Y, Zhang T, Qin Y, Hua D (2021) HIG1 Domain family member 1A disrupts proliferation, migration, and invasion of colon adenocarcinoma Cells. Bioengineered 12:10501–10511. 10.1080/21655979.2021.1999368 34787061 10.1080/21655979.2021.1999368 PMC8809935

[39] Jia L, Bickel JS, Wu J, Morgan MA, Li H, Yang J, Yu X, Chan RC, Sun Y (2011) RBX1 (RING Box Protein 1) E3 ubiquitin ligase is required for genomic integrity by modulating DNA replication licensing proteins. J Biol Chem 286:3379–3386. 10.1074/jbc.M110.188425 21115485 10.1074/jbc.M110.188425 PMC3030344

[40] Wang W, Qiu J, Liu Z, Zeng Y, Fan J, Liu Y, Guo Y (2013) Overexpression of RING Box Protein-1 (RBX1) associated with poor prognosis of non-muscle-invasive bladder transitional cell carcinoma. J Surg Oncol 107:758–761. 10.1002/jso.23317 23609182 10.1002/jso.23317

[41] Migita K, Takayama T, Matsumoto S, Wakatsuki K, Tanaka T, Ito M, Nishiwada S, Nakajima Y (2014) Prognostic impact of RING box protein-1 (RBX1) expression in gastric cancer. Gastric Cancer 17:601–609. 10.1007/s10120-013-0318-y 24292229 10.1007/s10120-013-0318-y

[42] Rudner AD, Hardwick KG, Murray AW (2000) Cdc28 activates exit from mitosis in budding yeast. J Cell Biol 149:1361–1376. 10.1083/jcb.149.7.1361 10871278 10.1083/jcb.149.7.1361 PMC2175138

[43] He J, Mao Y, Huang W, Li M, Zhang H, Qing Y, Lu S, Xiao H, Li K (2020) Methylcrotonoyl-CoA carboxylase 2 promotes proliferation, migration and invasion and inhibits apoptosis of prostate cancer cells through regulating GLUD1-P38 MAPK signaling pathway. OTT 13:7317–7327. 10.2147/OTT.S249906 10.2147/OTT.S249906 PMC7395692 32801758

[44] Liu Y, Yuan Z, Song C (2019) Methylcrotonoyl-CoA carboxylase 2 overexpression predicts an unfavorable prognosis and promotes cell proliferation in breast cancer. Biomark Med 13:427–436. 10.2217/bmm-2018-0475 30895811 10.2217/bmm-2018-0475

[45] Derenzini M (2002) Thymidylate synthase protein expression and activity are related to the cell proliferation rate in human cancer cell lines. Mol Pathol 55:310–314. 10.1136/mp.55.5.310 12354935 10.1136/mp.55.5.310 PMC1187262

[46] Kawabata S, Chiang C-T, Tsurutani J, Shiga H, Arwood ML, Komiya T, Gills JJ, Memmott RM, Dennis PA (2014) Rapamycin downregulates thymidylate synthase and potentiates the activity of pemetrexed in non-small cell lung cancer. Oncotarget 5:1062–1070. 10.18632/oncotarget.1760 24658085 10.18632/oncotarget.1760 PMC4011583

[47] Li Q, Zhu G-D (2002) Targeting serine / threonine protein kinase B / Akt and cell-cycle checkpoint kinases for treating cancer. CTMC 2:939–971. 10.2174/1568026023393318 10.2174/1568026023393318 12171565

[48] Sivaraman VS, Wang H, Nuovo GJ, Malbon CC (1997) Hyperexpression of mitogen-activated protein kinase in human breast cancer. J Clin Invest 99:1478–1483. 10.1172/JCI119309 9119990 10.1172/JCI119309 PMC507966

[49] Sofi S, Mehraj U, Qayoom H, Aisha S, Almilaibary A, Alkhanani M, Mir MA (2022) Targeting cyclin-dependent kinase 1 (CDK1) in cancer: molecular docking and dynamic simulations of potential CDK1 inhibitors. Med Oncol 39:133. 10.1007/s12032-022-01748-2 35723742 10.1007/s12032-022-01748-2 PMC9207877

[50] Guo R, Liu T, Shasaltaneh MD, Wang X, Imani S, Wen Q (2022) Targeting adenylate cyclase family: new concept of targeted cancer therapy. Front Oncol 12:829212. 10.3389/fonc.2022.829212 35832555 10.3389/fonc.2022.829212 PMC9271773

[51] Lv Q, Zhang J, Yi Y, Huang Y, Wang Y, Wang Y, Zhang W (2016) Proliferating cell nuclear antigen has an association with prognosis and risks factors of cancer patients: a systematic review. Mol Neurobiol 53:6209–6217. 10.1007/s12035-015-9525-3 26558632 10.1007/s12035-015-9525-3

[52] Wang L, Kong W, Liu B, Zhang X (2018) Proliferating cell nuclear antigen promotes cell proliferation and tumorigenesis by up-regulating STAT3 in non-small cell lung cancer. Biomed Pharmacother 104:595–602. 10.1016/j.biopha.2018.05.071 29803172 10.1016/j.biopha.2018.05.071

[53] Quéva C, McArthur GA, Ramos LS, Eisenman RN (1999) Dwarfism and dysregulated proliferation in mice overexpressing the MYC antagonist MAD1 10616903

[54] Hanif IM, Hanif IM, Shazib MA, Ahmad KA, Pervaiz S (2010) Casein kinase II: an attractive target for anti-cancer drug design. Int J Biochem Cell Biol 42:1602–1605. 10.1016/j.biocel.2010.06.010 20558317 10.1016/j.biocel.2010.06.010

[55] Greene JM, Feugang JM, Pfeiffer KE, Stokes JV, Bowers SD, Ryan PL (2013) L-Arginine enhances cell proliferation and reduces apoptosis in human endometrial RL95-2 cells. Reprod Biol Endocrinol 11:15. 10.1186/1477-7827-11-15 23442442 10.1186/1477-7827-11-15 PMC3598371

[56] Van Slegtenhorst M, Carr E, Stoyanova R, Kruger WD, Henske EP (2004) Tsc1+ and Tsc2+ regulate arginine uptake and metabolism in Schizosaccharomyces Pombe. J Biol Chem 279:12706–12713. 10.1074/jbc.M313874200 14718525 10.1074/jbc.M313874200

[57] Liu Y, Hyde AS, Simpson MA, Barycki JJ (2014) Emerging regulatory paradigms in glutathione metabolism. Adv Cancer Res 122:69–101 ISBN 978–0–12–420117–0 10.1016/B978-0-12-420117-0.00002-5 PMC4515967 24974179

[58] Hlozkova K, Thakker A, Alquézar-Artieda N, Zaliova M, Trka J, Tennant DA, Starkova J (2019) Separate roles of asparagine and glutamine in cytostatic effect of L-asparaginase - stable isotope tracing approach. Blood 134:2575–2575. 10.1182/blood-2019-127911

[59] Iiboshi Y, Papst PJ, Hunger SP, Terada N (1999) L-asparaginase inhibits the rapamycin-targeted signaling pathway. Biochem Biophys Res Commun 260:534–539. 10.1006/bbrc.1999.0920 10403802 10.1006/bbrc.1999.0920

[60] Song Q, Meng B, Xu H, Mao Z (2020) The emerging roles of vacuolar-Type ATPase-dependent lysosomal acidification in neurodegenerative diseases. Transl Neurodegener 9:17. 10.1186/s40035-020-00196-0 32393395 10.1186/s40035-020-00196-0 PMC7212675

[61] Li Y-Y, Qin Z-H, Sheng R (2024) The multiple roles of autophagy in neural function and diseases. Neurosci Bull 40:363–382. 10.1007/s12264-023-01120-y 37856037 10.1007/s12264-023-01120-y PMC10912456

[62] Neufeld TP (2010) TOR-dependent control of autophagy: biting the hand that feeds. Curr Opin Cell Biol 22:157–168. 10.1016/j.ceb.2009.11.005 20006481 10.1016/j.ceb.2009.11.005 PMC2854204

[63] Russell RC, Yuan H-X, Guan K-L (2014) Autophagy regulation by nutrient signaling. Cell Res 24:42–57. 10.1038/cr.2013.166 24343578 10.1038/cr.2013.166 PMC3879708

[64] Chen Y, Zheng Y, Foster DA (2003) Phospholipase D confers rapamycin resistance in human breast cancer cells. Oncogene 22:3937–3942. 10.1038/sj.onc.1206565 12813467 10.1038/sj.onc.1206565

[65] Park JB, Lee CS, Jang J-H, Ghim J, Kim Y-J, You S, Hwang D, Suh P-G, Ryu SH (2012) Phospholipase signalling networks in cancer. Nat Rev Cancer 12:782–792. 10.1038/nrc3379 23076158 10.1038/nrc3379

[66] Cavelius P, Engelhart-Straub S, Heieck K, Pilz M, Melcher F, Brück T (2022) Agricultural biocatalysis: from waste stream to food and feed additives. pp. 133–182 ISBN 978–1–00–331307–6

[67] Marsafari M, Xu P (2020) Debottlenecking mevalonate pathway for antimalarial drug precursor Amorphadiene biosynthesis in Yarrowia lipolytica. Metabol Eng Commun 10:e00121. 10.1016/j.mec.2019.e00121 10.1016/j.mec.2019.e00121 PMC6957783 31956504

[68] Seo Y, Kim J, Park SJ, Park JJ, Cheon JH, Kim WH, Kim TI (2020) Metformin suppresses cancer stem cells through AMPK activation and inhibition of protein Prenylation of the Mevalonate Pathway in Colorectal Cancer. Cancers 12:2554. 10.3390/cancers12092554 32911743 10.3390/cancers12092554 PMC7563617

[69] John Peter AT, Cheung NJ, Kornmann B (2022) Csf1: a putative lipid transport protein required for Homeoviscous adaptation of the lipidome. Contact 5:251525642211019. 10.1177/25152564221101974 10.1177/25152564221101974 PMC10243558 37366504

[70] Richardsen E, Uglehus RD, Johnsen SH, Busund L-T (2015) Macrophage-Colony Stimulating Factor (CSF1) predicts breast cancer progression and mortality. Anticancer Res 25667468

[71] Li Z, Liu H, Luo X. Lipid droplet and its implication in cancer progression PMC7783747 33414989

[72] Morimoto Y, Saitoh S, Takayama Y (2022) Growth conditions inducing G1 cell cycle arrest enhance lipid production in the Oleaginous yeast *Lipomyces Starkeyi*. J Cell Scie. 10.1242/jcs.259996 10.1242/jcs.259996 35833504

[73] Kohandel Z, Farkhondeh T, Aschner M, Pourbagher-Shahri AM, Samarghandian S (2022) Anti-inflammatory action of Astaxanthin and its use in the treatment of various diseases. Biomed Pharmacother 145:112179. 10.1016/j.biopha.2021.112179 34736076 10.1016/j.biopha.2021.112179

[74] Nishida Y, Nawaz A, Hecht K, Tobe K (2021) Astaxanthin as a novel mitochondrial regulator: a new aspect of carotenoids, beyond antioxidants. Nutrients 14:107. 10.3390/nu14010107 35010981 10.3390/nu14010107 PMC8746862

[75] Zhang L, Wang H (2015) Multiple mechanisms of anti-cancer effects exerted by astaxanthin. Mar Drugs 13:4310–4330. 10.3390/md13074310 26184238 10.3390/md13074310 PMC4515619

[76] Koklesova L, Liskova A, Samec M, Buhrmann C, Samuel SM, Varghese E, Ashrafizadeh M, Najafi M, Shakibaei M, Büsselberg D et al (2020) Carotenoids in cancer apoptosis—the road from bench to bedside and back. Cancers 12:2425. 10.3390/cancers12092425 32859058 10.3390/cancers12092425 PMC7563597

[77] Kotake-Nara E, Miyashita K, Nagao A, Kushiro M, Zhang H, Sugawara T (2001) Carotenoids affect proliferation of human prostate cancer cells. J Nutr 131:3303–3306. 10.1093/jn/131.12.3303 11739884 10.1093/jn/131.12.3303

[78] Pourzand C, Albieri-Borges A, Raczek NN (2022) Shedding a new light on skin aging, iron- and redox-homeostasis and emerging natural antioxidants. Antioxidants 11:471. 10.3390/antiox11030471 35326121 10.3390/antiox11030471 PMC8944509

[79] Varesi A, Chirumbolo S, Campagnoli LIM, Pierella E, Piccini GB, Carrara A, Ricevuti G, Scassellati C, Bonvicini C, Pascale A (2022) The role of antioxidants in the interplay between oxidative stress and senescence. Antioxidants 11:1224. 10.3390/antiox11071224 35883714 10.3390/antiox11071224 PMC9311946

[80] Diaconeasa Z, Știrbu I, Xiao J, Leopold N, Ayvaz Z, Danciu C, Ayvaz H, Stǎnilǎ A, Nistor M, Socaciu C (2020) Anthocyanins, vibrant color pigments, and their role in skin cancer prevention. Biomedicines 8:336. 10.3390/biomedicines8090336 32916849 10.3390/biomedicines8090336 PMC7555344

[81] Zhou X, Rao S, Wrightstone E, Sun T, Lui ACW, Welsch R, Li L (2022) Phytoene synthase: the key rate-limiting enzyme of carotenoid biosynthesis in plants. Front Plant Sci 13:884720. 10.3389/fpls.2022.884720 35498681 10.3389/fpls.2022.884720 PMC9039723

[82] Velayos A, Eslava AP, Iturriaga EA (2000) A bifunctional enzyme with lycopene cyclase and phytoene synthase activities is encoded by the *carRP* Gene of *Mucor Circinelloides*. Eur J Biochem 267:5509–5519. 10.1046/j.1432-1327.2000.01612.x 10951210 10.1046/j.1432-1327.2000.01612.x

[83] Werth EG, McConnell EW, Couso Lianez I, Perrine Z, Crespo JL, Umen JG, Hicks LM (2019) Investigating the Effect of target of rapamycin kinase inhibition on the *Chlamydomonas Reinhardtii* Phosphoproteome: from known homologs to new targets. New Phytol 221:247–260. 10.1111/nph.15339 30040123 10.1111/nph.15339

[84] Zhu Z, Ding Y, Gong Z, Yang L, Zhang S, Zhang C, Lin X, Shen H, Zou H, Xie Z et al (2015) Dynamics of the lipid droplet proteome of the oleaginous yeast *Rhodosporidium toruloides*. Eukaryot Cell 14:252–264. 10.1128/EC.00141-14 25576482 10.1128/EC.00141-14 PMC4346559

[85] Wang Y, Zhang S, Zhu Z, Shen H, Lin X, Jin X, Jiao X, Zhao ZK (2018) Systems analysis of phosphate-limitation-induced lipid accumulation by the oleaginous yeast *Rhodosporidium toruloides*. Biotechnol Biofuels 11:148. 10.1186/s13068-018-1134-8 29849765 10.1186/s13068-018-1134-8 PMC5968551

[86] Wang Y, Liu F, Liu H, Zhang Y, Jiao X, Ye M, Zhao ZK, Zhang S (2023) Regulation of autophagy and lipid accumulation under phosphate limitation in *Rhodotorula toruloides*. Front Microbiol 13:1046114. 10.3389/fmicb.2022.1046114 36777022 10.3389/fmicb.2022.1046114 PMC9908577

[87] Huang S, Houghton PJ (2001) Mechanisms of resistance to rapamycins. Drug Resist Updates 4:378–391. 10.1054/drup.2002.0227 10.1054/drup.2002.0227 12030785

[88] Nyfeler B, Bergman P, Triantafellow E, Wilson CJ, Zhu Y, Radetich B, Finan PM, Klionsky DJ, Murphy LO (2011) Relieving autophagy and 4EBP1 from rapamycin resistance. Mol Cell Biol 31:2867–2876. 10.1128/MCB.05430-11 21576371 10.1128/MCB.05430-11 PMC3133392

[89] Noh W-C, Mondesire WH, Peng J, Jian W, Zhang H, Dong J, Mills GB, Hung M-C, Meric-Bernstam F (2004) Determinants of rapamycin sensitivity in breast cancer cells. Clin Cancer Res 10:1013–1023. 10.1158/1078-0432.CCR-03-0043 14871980 10.1158/1078-0432.ccr-03-0043

[90] Bruemmer D, Yin F, Liu J, Kiyono T, Fleck E, Van Herle AJ, Law RE (2003) Rapamycin inhibits E2F-dependent expression of minichromosome maintenance proteins in vascular smooth muscle cells. Biochem Biophys Res Commun 303:251–258. 10.1016/S0006-291X(03)00343-7 12646195 10.1016/s0006-291x(03)00343-7

[91] Franco LC, Morales F, Boffo S, Giordano A (2018) CDK9: a key player in cancer and other diseases. J Cell Biochem 119:1273–1284. 10.1002/jcb.26293 28722178 10.1002/jcb.26293

[92] Jin YJ, Burakoff SJ (1993) The 25-kDa FK506-binding protein is localized in the nucleus and associates with casein kinase II and nucleolin. Proc Natl Acad Sci USA 90:7769–7773. 10.1073/pnas.90.16.7769 7689229 10.1073/pnas.90.16.7769 PMC47224

[93] Laplante M, Sabatini DM (2009) mTOR signaling at a glance. J Cell Sci 122:3589–3594. 10.1242/jcs.051011 19812304 10.1242/jcs.051011 PMC2758797

[94] Minarowska A Regulatory role of cathepsin D in apoptosis 17951163

[95] Repalli J (2015) Translocator protein (TSPO) role in aging and Alzheimer’s disease. CAS 7:168–175. 10.2174/1874609808666141210103146 10.2174/1874609808666141210103146 PMC4435228 25495567

[96] Barczyk K, Kreuter M, Pryjma J, Booy EP, Maddika S, Ghavami S, Berdel WE, Roth J, Los M (2005) Serum cytochrome c indicates *in Vivo* apoptosis and can serve as a prognostic marker during cancer therapy. Intl J Cancer 116:167–173. 10.1002/ijc.21037 10.1002/ijc.21037 15800951

[97] Basnakian AG, Apostolov EO, Yin X, Abiri SO, Stewart AG, Singh AB, Shah SV (2006) Endonuclease G promotes cell death of non-invasive human breast cancer cells. Exp Cell Res 312:4139–4149. 10.1016/j.yexcr.2006.09.012 17046751 10.1016/j.yexcr.2006.09.012 PMC1839947

[98] Li LY, Luo X, Wang X (2001) Endonuclease G is an apoptotic dnase when released from mitochondria. Nature 412:95–99. 10.1038/35083620 11452314 10.1038/35083620

[99] Aman Y, Schmauck-Medina T, Hansen M, Morimoto RI, Simon AK, Bjedov I, Palikaras K, Simonsen A, Johansen T, Tavernarakis N et al (2021) Autophagy in healthy aging and disease. Nat Aging 1:634–650. 10.1038/s43587-021-00098-4 34901876 10.1038/s43587-021-00098-4 PMC8659158

[100] Russell RC, Guan K (2022) The multifaceted role of autophagy in cancer. EMBO J 41:e110031. 10.15252/embj.2021110031 35535466 10.15252/embj.2021110031 PMC9251852

[101] Debnath J, Gammoh N, Ryan KM (2023) Autophagy and autophagy-related pathways in cancer. Nat Rev Mol Cell Biol 24:560–575. 10.1038/s41580-023-00585-z 36864290 10.1038/s41580-023-00585-z PMC9980873

[102] Li X, He S, Ma B (2020) Autophagy and autophagy-related proteins in cancer. Mol Cancer 19:12. 10.1186/s12943-020-1138-4 31969156 10.1186/s12943-020-1138-4 PMC6975070

[103] Wong SQ, Kumar AV, Mills J, Lapierre LR (2020) Autophagy in aging and longevity. Hum Genet 139:277–290. 10.1007/s00439-019-02031-7 31144030 10.1007/s00439-019-02031-7 PMC6884674

[104] Engelhart-Straub S, Cavelius P, Hölzl F, Haack M, Awad D, Brueck T, Mehlmer N (2022) Effects of light on growth and metabolism of rhodococcus erythropolis. Microorganisms 10:1680. 10.3390/microorganisms10081680 36014097 10.3390/microorganisms10081680 PMC9416670

[105] Kanehisa M, Sato Y, Morishima K (2016) BlastKOALA and GhostKOALA: KEGG tools for functional characterization of genome and metagenome sequences. J Mol Biol 428:726–731. 10.1016/j.jmb.2015.11.006 26585406 10.1016/j.jmb.2015.11.006

[106] Polyansky A, Shatz O, De EZ (2020) *Novo* phospholipid synthesis promotes efficient autophagy. Biochemistry 59:1011–1012. 10.1021/acs.biochem.0c00115 32119532 10.1021/acs.biochem.0c00115

[107] Tran NH, Zhang X, Xin L, Shan B, De LM (2017) Novo peptide sequencing by deep learning. Proc Natl Acad Sci USA 114:8247–8252. 10.1073/pnas.1705691114 28720701 10.1073/pnas.1705691114 PMC5547637

