# Supplementary material for: Rhodosporidium toruloides—a new surrogate model to study rapamycin induced effects on human aging and cancer
Source: Cell Mol Life Sci. 2025 Apr 9;82(1):153. doi: 10.1007/s00018-025-05662-4 (PMC11982011; doi:10.1007/s00018-025-05662-4)

Supplementary Material

Cellular and Molecular Life Sciences

*Rhodosporidium toruloides* - a new surrogate model to study rapamycin induced effects on human aging and cancer

Philipp M. Cavelius^1^, Martina Haack^1^, Dania Awad^1^, Thomas B. Brueck^1,*^ and Norbert Mehlmer^1,*^

^1^ Werner Siemens-Chair of Synthetic Biotechnology, Department of Chemistry, Technical University of Munich (TUM), Garching, Germany; brueck@tum.de; norbert.mehlmer@tum.de

***** Correspondence: brueck@tum.de; [norbert.mehlmer@tum.de](mailto:norbert.mehlmer@tum.de)

ORCID:

PC: 0000-0003-1774-149X

DA: 0000-0003-0339-399X

TB: 0000-0002-2113-6957

NM: 0000-0002-6854-4341

**Keywords:** haplotypes/Rhodosporidium/rapamycin/target of rapamycin/time-resolved proteomics

**Turbidity and Coloration**

Throughout the cultivation period, distinct differences in total carotenoid accumulation were observed between rapamycin treated samples and non-treated samples identified by changes in coloration and turbidity (Figure S1). It became evident, that in YPD medium, rapamycin induced early coloration, suggesting an increase in carotenoid levels, cell densities or both. However, in later stages, colorations of untreated samples appeared to approximate those of rapamycin cultures. Furthermore, IFO0880 cultures subjected to rapamycin appeared to grow significantly slower in the early stages of cultivation as can be estimated on basis of turbidity. In YNB medium, visible differences in coloration or turbidity were minimal.

*
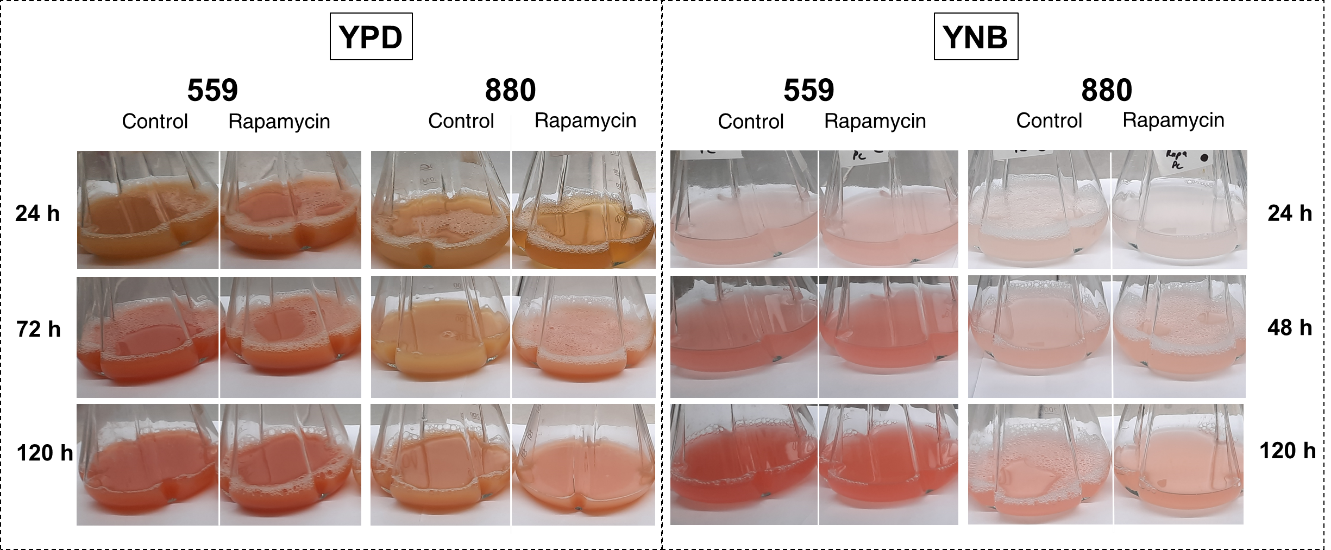
*

**Figure S1: Changes in turbidity and coloration between haplotypes, media and time points.**

**Changes in fatty acid profile**

Figure S2 depicts all changes recorded in fatty acid profile for cultures grown in YNB or YPD medium with and without addition of rapamycin. Consistent to our previous reporting (Cavelius *et al*, 2023), oleic acid (C18:1) represents the major fatty acid across all samples (between 31.5% and 64.7%), followed by linoleic acid (C18:2), stearic acid (C18:0) and palmitic acid (C16:0), which make up large amounts of the total fatty acid content throughout all samples. Interestingly, in YNB grown samples, C18:0 levels seem to be elevated in comparison to their respective YPD counterparts. Furthermore, C17:0 accumulates to higher levels in YNB samples than in corresponding YPD samples. Similar to previous studies (Cavelius *et al*, 2023), oleic acid levels decrease over time in IFO0559 samples grown in YNB. C18:1 levels measure 36.3% at 48 h decreasing to 31.5% after 120 h. Interestingly, rapamycin seems to stabilize intracellular levels of oleic acid, showing levels in IFO0559 of 43.9% and 45.7% at 48 h and 120 h, respectively. In IFO0880 C18:1 levels slightly increase when cultivated in YNB from 41.1% to 46.6%, with further increase in rapamycin treated samples compared to untreated controls. In YPD medium, effects are similar, with IFO0559 oleic acid levels decreasing from 64.7% to 60.3%, while IFO0880 C18:1 levels of untreated samples increase from 57.9% at 48 h to 61.1% at 120 h. Interestingly, rapamycin treatment of YPD grown cultures led to a decrease in oleic acid, in both haplotypes of *R. toruloides* at 48 h. In IFO0559 C18:1 content drops from 64.7% to 59.4 % and in IFO0880 a decrease from 57.9% to 40.0% was observed. At the same time, levels of C18:2 and C18:3 were increased.


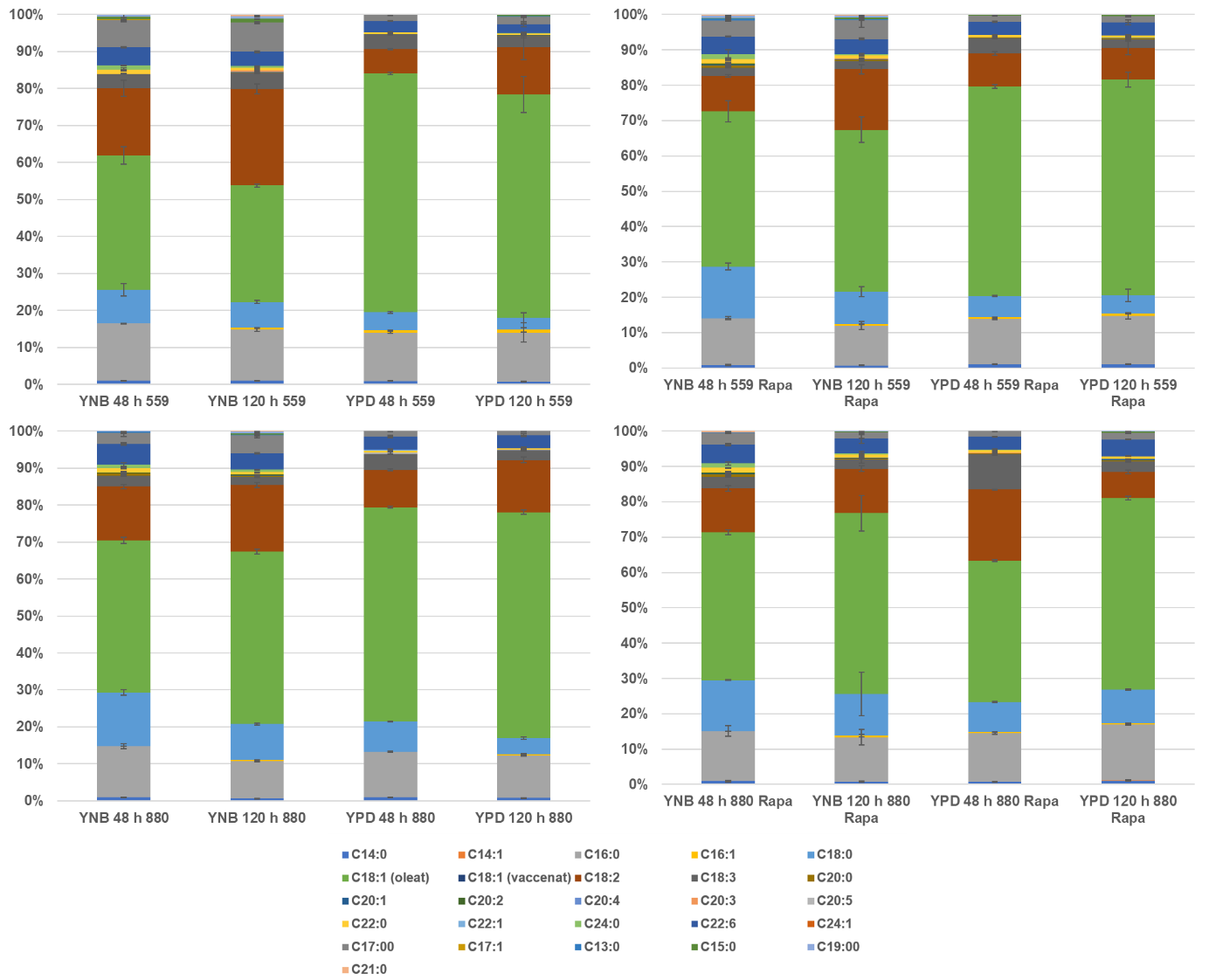


**Figure S2**. Relative composition of fatty acids in IFO0559 and IFO0558 at 48 h and 120 h, in YNB and YPD with and without addition of rapamycin.

**TOR kinase Sequence analysis**

As growth and proteomics data suggests some kind of reduced sensitivity of IFO0559 to rapamycin compared to IFO0880. TOR kinase protein sequences were compared for mutations.

15 mutation sites between the haplotypes were identified through comparison of TOR kinase amino acid sequences (Table S3). Where possible, sequences were aligned (local alignment Smith-Waterman) and compared to human TOR kinase. Among the mutations, 2 Insert mutations identified in IFO0559 were neither found in IFO0880 nor in the correlating sequence of human TOR kinase (Figure S4). Furthermore, L1058 of IFO0880 was conserved between all variants except IFO0559, where it was replaced with a histidine. However, these mutations were not positioned in sequences previously described to impact rapamycin sensitivity when mutated. Nonetheless, they might impact rapamycin sensitivity through changes in protein tertiary structure.

**Table S3.** Amino acid mutation analysis TOR kinase *R. toruloides* IFO0880, IFO0559, human and *S. cerevisae*.

| **aa position in IFO0880** | **aa (IFO0880)** | **aa (IFO0559)** | **aa human** |
| --- | --- | --- | --- |
| 44 | N | S | Q |
| 488 - 499 | no insert | 16 aa Insert | no insert |
| 697 | I | T | R |
| 783 | T | I | K |
| 785 | V | I | N |
| 955 | Q | H | H |
| 1014 | V | I | V |
| 1058 | L | H | L |
| 1109 - 1113 | CVV | 3 aa deletion | not identified |
| 1431 | H | Q | C |
| 1486 | V | A | E |
| 1604 | S | P | T |
| 1650 | L | I | I |
| 2093 - 2094 | no insert | 4aa insert | no insert |
| 2275 | D | G | H |

**Figure S4:** Amino acid sequence alignements IFO0559 TOR kinase insert mutations to IFO0880 and human TOR kinase.


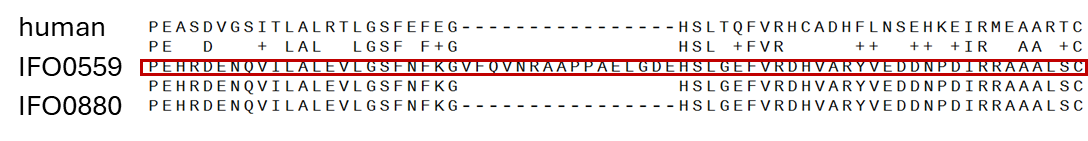

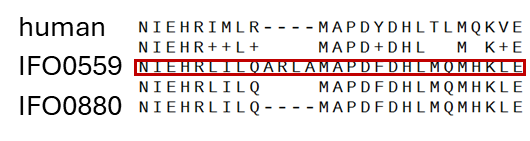

Supplement: Supplementary file 1 — Supplementary file1 (DOCX 1362 KB) [file 18_2025_5662_MOESM1_ESM.docx]
